# Spheroid size influences cellular senescence and angiogenic potential of mesenchymal stromal cell-derived soluble factors and extracellular vesicles

**DOI:** 10.3389/fbioe.2023.1297644

**Published:** 2023-12-12

**Authors:** Matteo Rovere, Daniele Reverberi, Pietro Arnaldi, Maria Elisabetta Federica Palamà, Chiara Gentili

**Affiliations:** ^1^ Department of Experimental Medicine, University of Genoa, Genoa, Italy; ^2^ IRCCS Ospedale Policlinico San Martino, Genoa, Italy

**Keywords:** mesenchymal stromal cells, spheroids, senescence, secretome, extracellular vesicles, angiogenesis

## Abstract

**Introduction:** The secretome of mesenchymal stromal cells (MSCs) serves as an innovative tool employed in the regenerative medicine approach. In this particular context, three-dimensional (3D) culture systems are widely utilized to better replicate *in vivo* conditions and facilitate prolonged cell maintenance during culture. The use of spheroids enables the preservation of the classical phenotypical characteristics of MSCs. However, the distinct microenvironment within the spheroid may impact the secretome, thereby enhancing the angiogenic properties of adult MSCs that typically possess a reduced angiogenic potential compared to MSCs derived from perinatal tissues due to the hypoxia created in the internal region of the spheroid.

**Methods:** In this study, large spheroids (2,600 cells, ∼300 μm diameter) and small spheroids (1,000 cells, ∼200 μm diameter) were used to examine the role of spheroid diameter in the generation of nutrients and oxygen gradients, cellular senescence, and the angiogenic potential of secreted factors and extracellular vesicles (EVs).

**Results:** In this study, we demonstrate that large spheroids showed increased senescence and a secretome enriched in pro-angiogenic factors, as well as pro-inflammatory and anti-angiogenic cytokines, while small spheroids exhibited decreased senescence and a secretome enriched in pro-angiogenic molecules. We also demonstrated that 3D culture led to a higher secretion of EVs with classical phenotypic characteristics. Soluble factors and EVs from small spheroids exhibited higher angiogenic potential in a human umbilical vein endothelial cell (HUVEC) angiogenic assay.

**Discussion:** These findings highlighted the necessity of choosing the appropriate culture system for obtaining soluble factors and EVs for specific therapeutic applications.

## 1 Introduction

The secretion of paracrine factors and extracellular vesicles (EVs) by mesenchymal stromal cells (MSCs) has gained a lot of attention in the field of regenerative medicine (Rani et al., 2015; Ferreira et al., 2018; Praveen Kumar et al., 2019; Rezaie et al., 2019). MSCs are largely known for their immunomodulatory, anti-inflammatory, and anti-microbial effects (Kyurkchiev et al., 2014; Fierabracci et al., 2016; Marrazzo et al., 2019; Markov et al., 2021). However, their ability to stimulate angiogenesis has also been widely reported (Watt et al., 2013). MSCs indeed produce and secrete a plethora of pro-angiogenic factors, such as angiogenin, vascular endothelial growth factor-A (VEGF-A), interleukin-8 (IL-8), and hepatocyte growth factor (HGF), able to promote new vessel formation (Burlacu et al., 2013; Liu et al., 2015). Interestingly, MSCs isolated from different sources possess different angiogenic potentials. More deeply, MSCs obtained from the amniotic fluid and umbilical cord showed better support for the angiogenic process than bone marrow-derived MSCs, suggesting that MSCs from early developmental stages are more supportive than adult MSCs (Roura et al., 2012; Roubelakis et al., 2013; Pinto et al., 2020).

The limited angiogenic potential surely presents a limitation for the use of adult MSCs, but the most relevant constraint is related to the onset of cellular senescence during the long-term expansion required to obtain a clinically relevant cell amount. Cellular senescence is characterized by cell proliferation blockage, altered cell morphology (Neurohr et al., 2019), increased DNA damage, and a change in gene expression and a secretory profile known as the senescence-associated secretory phenotype (SASP). This is characterized by the high secretion of pro-inflammatory cytokines, reduced secretion of cytokines with immunomodulatory and anti-inflammatory effects, and reduced production of EV (Coppé et al., 2010; Turinetto et al., 2016; Jeske et al., 2021). Specifically, SASP is characterized by the upregulation of different pro-inflammatory cytokines, metalloproteases, and growth factors (Acosta et al., 2013; Lunyak et al., 2017). The activation of cellular senescence in *in vitro* cultures is usually related to the onset of replicative senescence, which can be caused by a plethora of factors, such as telomere shortening, DNA damage, and oxidative stress (Wagner et al., 2008; Pizzuti et al., 2023). Analysis of the cellular senescence of MSCs *in vitro* is extremely important for controlling the quality of cells used in cellular therapy approaches. It has been widely observed that the onset of replicative senescence may lead to a complete alteration of MSC characteristics and a reduction of their regenerative potential. In addition, MSC senescence has been proved to impair the immunomodulatory and anti-inflammatory abilities of MSCs (Meng et al., 2017).

Three-dimensional (3D) culture methods represent an alternative strategy that could potentially overcome the major limitations of classical two-dimensional (2D) cultures. These systems could better recapitulate the *in vivo* conditions, allowing them to recreate a more physiological environment with proper cell–cell and cell–matrix interactions (Soares et al., 2012; Costa et al., 2016; Langhans, 2018). Among 3D culture methods, spheroids are most widely used in cancer research, but they also represent a promising approach in other research fields, such as stem cells and regenerative medicine. Spheroid cultures of MSCs have indeed already been reported to allow more efficient cell organization and enhance the therapeutic potential of MSCs (Chen et al., 2021; Kouroupis and Correa, 2021). For instance, it has been demonstrated that MSCs cultured as spheroids possess enhanced immunomodulatory characteristics as well as increased anti-inflammatory effects (Regmi et al., 2021; Xie et al., 2021). 3D architecture also supports a more *in vivo*-like cell behavior, leading to a reorganization of the cell junction (i.e., with the expression of E-cadherin) and cytoskeleton and a drastic change in cell morphology (Cesarz and Tamama, 2016). Furthermore, some authors demonstrated that MSC spheroid cultures can stimulate an increase in cell viability (Regmi et al., 2021), differentiation capability, and regenerative potential (Ohori-Morita et al., 2022), sustained by a delay of replicative senescence (Cesarz and Tamama, 2016).

However, spheroid culture also presents some limitations due to the generation of different regions inside the aggregates. More specifically, the outer region, being exposed to external nutrients, is mostly populated by proliferating cells, the intermediate region hosts senescent cells, and the internal region becomes a necrotic and apoptotic core of cells due to the establishment of nutrients and oxygen gradients, causing cell suffering (Mueller-Klieser, 1984; Alvarez-Pérez et al., 2005; Curcio et al., 2007; Kouroupis and Correa, 2021). Several studies demonstrated that the control of spheroid diameter is crucial in the generation of these gradients, and a diameter of 200 μm was set as the threshold for the generation of the internal hypoxic core (Alvarez-Pérez et al., 2005; Curcio et al., 2007; Bhang et al., 2011). However, the formation of mild nutrients and oxygen gradients always occurs even in small-diameter spheroids. This process could work as internal priming for the cells, leading to an enhancement of the angiogenic properties of MSC-derived soluble factors and EVs (Cheng et al., 2013; Costa et al., 2017; Redondo-Castro et al., 2018; Ezquerra et al., 2021). The standardized production of spheroids with similar dimensions and characteristics is of extreme importance for their use in regenerative medicine. Moreover, the possible use of label-free methods for characterization, such as mass density analysis, could be an extremely useful system to determine the density and mass of the spheroid since alteration may suggest different secretory characteristics (Marrazzo et al., 2021).

The present study aims to demonstrate the hypothesis that spheroid diameter is crucial for the regulation of cell senescence and secretome composition, as well as for EV secretion. For this purpose, bone marrow-derived human MSCs were used to produce large- and small-sized spheroids. Cell properties, such as stemness and senescence, were analyzed to evaluate whether spheroid size might influence cell behavior. Moreover, the pro-angiogenic activity of secretome and EVs was evaluated on human umbilical vein endothelial cells (HUVECs).

## 2 Materials and methods

### 2.1 Mesenchymal stromal cell isolation and culture

MSCs were isolated from bone marrow aspirates derived from patients undergoing total or partial arthroscopy after obtaining informed consent (CER Liguria: 372/2019). In brief, bone marrow was washed with phosphate-buffered saline (PBS) and centrifuged at 300* g* for 10 min. Nucleated cells were seeded at a density of 1.5 × 10^5^ cells/cm^2^ and cultured in α-MEM GlutaMAX medium (Gibco, Waltham, MA, United States) supplemented with 10% fetal bovine serum (FBS) (Gibco), 100 U/mL penicillin/streptomycin (Euroclone, Milan, Italy), and 1 ng/mL fibroblast growth factor 2 (FGF-2) (PrepoTech) (complete medium). Cells were maintained in a humidified incubator at 37°C and 5% CO_2_. After 5 days, they were washed with PBS to remove unattached cells and a fresh, complete medium was added. At 90% confluence, cells were detached using trypsin/EDTA (Euroclone) and used for further experiments. Only cells at early passages (passage 2) were used for the subsequent analyses. For all the experiments, MSCs were cultured as spheroids and monolayer culture was used as a control.

### 2.2 Spheroid formation and culture

Spheroids were formed using a non-adherent MSC culture that stimulates MSC aggregation. 3D Petri Dish^®^ molds (MicroTissues Inc.) were used following the manufacturer’s instructions. 3D Petri dish molds, containing 96 circular wells with a depth of 800 µm and a diameter of 400 μm, were placed on a 24-well plate and conditioned for 24 h in a complete medium. After conditioning, either 2.5 × 10^5^ or 1 × 10^5^ cells re-suspended in 75 μL of the complete medium were seeded into the mold, allowing spheroid formation of approximately 2,600 cells (large spheroids) and 1,000 cells (small spheroids); 10 min after the seeding of the cells, 1 mL of the additional complete medium was added outside the 3D Petri dish. Spheroids were maintained in culture for 72 h prior to being used for further analyses. Spheroids were monitored daily under the microscope, and images were acquired using a Leica DMi1 microscope (Leica Microsystem, Wetzlar, Germany). Changes in spheroid diameter over time were measured using ImageJ software.

### 2.3 MSC phenotypic characterization

The surface MSC phenotype was analyzed using flow cytometry analysis. 3D Petri dishes were inverted and centrifuged at 300* g* for 10 min to remove spheroids from the agar. Spheroids and 2D MSCs were then trypsinized using 2.5% Trypsin (Gibco) for 15 min, and 50,000 cells/tube were incubated with specific primary antibodies conjugated with fluorescein isothiocyanate (FITC) or phycoerythrin (PE): FITC-anti-human CD31, FITC-anti-human CD34 (BD Bioscience, San Diego, CA, United States), PE-anti-human CD90, and PE-anti-human CD105 (BD Pharmingen, San Diego, CA, United States). Cells were also stained with the correlate IgG isotype controls conjugated with FITC (BD Bioscience) or PE (BD Pharmingen). Stainings were performed for 30 min at room temperature (RT) in the dark. Samples were run on a CytoFLEX Flow Cytometer (Beckman Coulter, Brea, CA, United States), and data were analyzed using FlowJo software (BD Biosciences).

### 2.4 Colony-forming unit (CFU) assay

After trypsinization, MSCs derived from both monolayer and spheroid cultures were seeded on a 10-cm-diameter Petri dish at a density of 10 cells/cm^2^ (Banfi et al., 2000). Cells were maintained in culture for 15 days in the complete medium, with refreshment every 3 days. Cells were then fixed in 10% buffered formalin (Bio Optica, Milan, Italy) for 10 min and stained with 1% methylene blue for 45 min. Stained dishes were scanned using the Epson Perfection 1260 scanner, and colony-forming units (CFUs) were analyzed using the ImageJ ColonyArea plugin (Guzmán et al., 2014). Colonies’ cell morphology was evaluated using a Leica DMi1 inverted phase microscope (Leica).

### 2.5 Viability analysis

A total of 20,000 cells were seeded on a glass coverslip for 2D MSC culture, and MSC spheroids were obtained as described above. Cell viability was assessed using a LIVE/DEAD^®^ imaging assay (Thermo Fisher Scientific), following the manufacturer’s instructions. Images were acquired using an AxioPhot microscope (Carl Zeiss, Jena, Germany, for 2D cultured MSCs) and an EVOS FL microscope (Thermo Fisher Scientific, for 3D spheroids) and analyzed using ImageJ software.

### 2.6 Immunofluorescence staining

Expression of HIF-1α, VEGF-A, Ki-67, cleaved caspase-3, and p21 in large and small spheroids was assessed via immunofluorescence. For 2D MSC culture, 20,000 cells were seeded on a glass coverslip, and MSC spheroids were obtained as described above. For cleaved caspase-3, a positive control was performed by treating monolayer MSCs with 10 µM etoposide for 72 h to verify antibody specificity. After 72 h of culture, microtissues were fixed in 10% buffered formalin for 10 min, embedded in OCT (Bio Optica), and stored at −80°C. Cryosections of 8 μm thickness were obtained using a Leica Cryostat. Sections were permeabilized in 0.5% Triton X-100 in PBS for 15 min. Unspecific binding was prevented by blocking the samples with 3% bovine serum albumin (BSA)/0.5% Triton X-100 in PBS for 1 h at room temperature. Sections were incubated overnight at 4°C with specific primary antibodies directed against HIF-1α (dilution 1:500, Santa Cruz Biotechnology), VEGF-A (dilution 1:500, Santa Cruz Biotechnology), cleaved caspase-3 (dilution 1:200, Cell Signaling Technology), Ki-67 (dilution 1:6400, Cell Signaling Technology), and p21 (dilution 1:800, Cell Signaling Technology); all antibodies were diluted in 1.5% BSA/0.5% Triton X-100 in PBS. After several washes in PBS/0.5% Triton X-100, samples were incubated with specific secondary goat anti-rabbit/mouse antibodies, conjugated with Alexa Fluor 488 (dilution 1:300, Life Technologies), and diluted in 1.5% BSA/0.5% Triton X-100 for 75 min at room temperature. The cytoskeleton was visualized with 5 U/mL phalloidin-594 staining (Life-Tech). Cell nuclei were stained with DAPI (dilution 1:10,000, Thermo Fisher Scientific) for 5 min at room temperature, and slides were cover-slipped with an aqueous mounting medium. Negative controls were run in parallel for all the staining by incubating the sections only with secondary antibodies to exclude any unspecific binding. Images were acquired using an Apotome microscope (Carl Zeiss) and analyzed using ImageJ software. The mean diameter of cryosections was also calculated, and only cryosections of the inner region of the spheroids were considered for analysis, while those of the outer region were excluded.

### 2.7 RNA extraction and quantitative real-time polymerase chain reaction

Total RNA from both MSC monolayer and spheroid cultures was collected after 72 h of culture using TRIzol™ reagent (Thermo Fisher Scientific), following the manufacturer’s instructions. The RNA concentration was quantified by measuring absorbance at 260 nm using a spectrophotometer (BioSpectrometer, Eppendorf, Milan, Italy). RNA purity was checked considering the 260 nm/280 nm ratio, with values included from 1.5 to 2.1. Complementary DNA (cDNA) was synthesized starting from 2 μg of total RNA using the SuperScript™ VILO™ Master Mix (Thermo Fisher Scientific), following the manufacturer’s instructions. Transcript levels of target genes (*CDK1*, *HIF-1α*, *LAMB1*, and *p21*) were measured using quantitative real-time polymerase chain reaction (qRT-PCR) performed on a 7500 Fast Real-Time PCR System (Applied Biosystems, Waltham, MA, United States) using the SYBR Green Master Mix (BrightGreen 2X qPCR, ABM^®^, Richmond, Canada). Sequence of primer used to evaluate the expression of the target genes are listed in Table 1. The housekeeping gene *GAPDH* was used as an endogenous control for normalization. Data were analyzed using the ΔΔCt method, and the results were shown as fold changes of the target gene with respect to a 2D condition normalized to the fold change of *GAPDH*.

**TABLE 1:** Primers used for quantitative real-time PCR.

Gene	Forward primer	Reverse primer
*CDK1*	5′-TTC​CTC​TCC​AAA​ATG​CCA​GA-3′	5′-AGG​GcG​GaT​TGG​AAA​TGA​AC-3′
*GAPDH*	5′-CCA​TCT​TCC​AGG​AGC​GAG​AT-3′	5′-CTG​CTT​CAC​CAC​CTT​CTT​GAT-3′
*HIF-1α*	5′-AGG​AAT​TAT​TTA​GCA​TGT​AGA​CTG​CTG​G-3′	5′-CAT​AAC​TGG​TCA​GCT​GTG​GTA​ATC​C-3′
*LAMB1*	5′-AAG​CAG​CTG​GAG​TGG​TTG​TT-3′	5′-TTG​GAT​GCT​CTT​GGG​GTT​C-3′
*P21*	5′-TCA​CTG​TCT​TGT​ACC​CTT​GTG​C-3′	5′-GGC​GTT​TGG​AGT​GGT​AGA​AA-3′

### 2.8 Cell senescence evaluation: Sudan black B staining and senescence-associated β-galactosidase activity

Senescence levels were evaluated using Sudan black B (SBB) staining, a dye capable of labeling lipofuscin (Evangelou and Gorgoulis, 2017). For 2D MSC cultures, 20,000 cells were seeded on a glass coverslip, and MSC spheroids were obtained as described above. Positive control was performed by treating MSCs with 20 μM H_2_O_2_ for 2 h, and treated cells were then maintained in the complete medium for 24 h before staining (Kiyoshima et al., 2012). Both coverslips and MSC spheroids were fixed in 10% buffered formalin for 10 min, and spheroid cryosections were obtained, as previously explained. Sections and coverslips were dehydrated in 70% ethanol and stained with a 0.7% SBB solution (Evangelou and Gorgoulis, 2017). Cell nuclei were counterstained using a 0.1% nuclear fast red solution. Slides and coverslips were mounted with a glycerol mounting medium (Dako, Santa Clara, CA, United States). β-Galactosidase activity was assessed using a Senescence β-Galactosidase Staining Kit (Cell Signaling Technology). In particular, for spheroid staining, microtissues were fixed for 10 min using the solution provided with the kit (glutaraldehyde, methanol, and formaldehyde), and spheroid cryosections were obtained as previously explained. Final staining was performed on both cryosections and coverslips, following the manufacturer’s instructions. Coverslips were mounted using an aqueous mounting medium (Dako). For spheroid cryosections, cell nuclei were stained with Mayer’s hematoxylin (Sigma-Aldrich) for 5 min at room temperature, and slides were cover-slipped with an aqueous mounting medium. Images were acquired using an AxioPhot microscope (Carl Zeiss) and analyzed using ImageJ software. The mean diameter of cryosections was also calculated, and only cryosections of the inner region of the spheroids were considered for analysis, while cryosections of the outer region were excluded.

### 2.9 EV separation

After 72 h of seeding, MSC cultures were rinsed twice with PBS and maintained for 30 min in α-MEM GlutaMAX medium supplemented with only 100 U/mL penicillin/streptomycin (serum-free medium, SF). Medium was replaced with fresh SF medium, and cells were maintained in an incubator at 37°C with 5% CO_2_ for 24 h. After cell pre-conditioning, conditioned medium (CM) were collected and centrifuged at 300* g* for 10 min at 4°C to remove cells and cell debris and at 2000* g* for 20 min at 4°C to remove apoptotic bodies. CM were concentrated using Amicon Ultra Filters with a molecular weight cut-off of 100 KDa (Millipore, Burlington, MA, United States). Concentrated CM were centrifuged at 10,000* g* for 40 min at 4°C to pellet the fraction of EVs enriched in medium-sized vesicles (mEVs). The supernatant was centrifuged at 100,000* g* for 2 h at 4°C to pellet the fraction of EVs enriched in small-sized vesicles (sEVs). Supernatants derived from 100,000* g* centrifugation were collected as depleted medium (DM). EV pellets were washed with PBS and centrifuged with the same *g* acceleration and time used for their separation at 4°C. After the washing step, EV pellets were re-suspended in 50 μL of 0.22-μm-filtered PBS for the subsequent analysis. A Beckman Coulter ultracentrifuge (Beckman Coulter Optima XPN-100 ultracentrifuge, Beckman Coulter) was used with swinging bucket rotors of type SW55Ti. The concentration of membrane-bound proteins of isolated EVs, used to indirectly quantify EV concentration and protein amount of CM and DM, was evaluated using a bicinchoninic acid assay (BCA), following the manufacturer’s instructions (Thermo Fisher Scientific). The total protein content was normalized to the initial number of seeded cells.

### 2.10 Protein composition analysis of MSC-derived conditioned medium

Analysis of protein composition was performed using the Proteome Profiler Human XL Cytokine Array Kit (R&D Systems), following the manufacturer’s instructions. In brief, 15 μg of proteins of CM were incubated with a cocktail of antibodies conjugated to the surface of a membrane overnight at 4°C. After several washing steps, the membrane was incubated with a cocktail of biotin-conjugated antibodies for 1 h at room temperature, then with HRP-conjugated streptavidin for 30 min at room temperature. After treatment of the membrane with substrate necessary for the HRP reaction (luminol and hydrogen peroxide) (LiteAblot^®^ TURBO, Euroclone), chemiluminescence was impressed on autoradiographic film (Fujifilm). Membranes were scanned using the Epson Perfection 1260 scanner, and analysis was performed using ImageJ to detect the pixel intensity. Four independent experiments were performed. Intensity values were normalized to the control spots of the membrane. Gene Ontology (GO) was performed on proteins with a normalized pixel intensity ≥0.3 using DAVID, focusing on the “*Biological process (BP)*” term. Graphical representations of cytokine array analysis and GO were carried out using R libraries: pheatmap, ggplot2, and circlize (Gu et al., 2014), setting the adjusted *p-*value < 0.05 as significant.

### 2.11 VEGF quantification on conditioned medium

Conditioned medium for MSCs derived from the three different culture conditions were collected, and VEGF was quantified via enzyme-linked immunosorbent assay (ELISA) using the Human VEGF ELISA Kit (Thermo Fisher Scientific), following the manufacturer’s instructions. Data were normalized to the initial number of seeded cells.

### 2.12 Western blot analysis

For Western blot analysis, after separation, mEVs and sEVs were re-suspended in 50 μL of RIPA buffer (1% Nonidet P-40, 0.1% sodium deoxycholate, and 0.1% sodium dodecyl sulfate in PBS, pH 7.5) supplemented with protease inhibitors (Cell Signaling Technology, Danvers, MA, United States). Cell lysates, used as controls, were obtained by lysing 60,000 MSCs for monolayer culture and one microtissue for each condition in 100 μL of RIPA buffer supplemented with protease inhibitors. After protein quantification with the BCA assay, electrophoresis was run by loading 1 μg of proteins onto 4%–12% Tris-Glycine gels (Invitrogen), and proteins were finally transferred on a PVDF membrane (Millipore). After blocking with 5% non-fat dry milk in TTBS (500 mM NaCl, 0.1% v/v Tween, and 20 mM Tris, pH 7.5) for 1 h at room temperature, membranes were incubated overnight at 4°C with specific primary antibodies diluted in 2.5% non-fat dry milk in TTBS and directed against syntenin-1 (dilution 1:1000, ab133267, Abcam), flotillin-1 (dilution 1:10,000, ab133497, Abcam, Cambridge, United Kingdom), CD63 (dilution 1:500, 10628D, Invitrogen, Waltham, MA, United States), CD81 (dilution 1:1000, 555675, BD Biosciences), Grp94 (dilution 1:1000, ab3674, Abcam), and calnexin (dilution 1:1000, Abcam). After several washes in TTBS, membranes were incubated with specific HRP-conjugated goat anti-rabbit/mouse antibodies diluted in 2.5% non-fat dry milk in TTBS (dilution 1:2000, Cell Signaling Technology, Danvers, MA, United States) for 1 h at room temperature. The detection of proteins was performed by providing the substrate for the HRP reaction (LiteAblot^®^ TURBO, Euroclone), and chemiluminescence was impressed on autoradiographic film (Fujifilm, Tokyo, Japan). Images were scanned using the Epson Perfection 1260 scanner.

### 2.13 Nanoparticle tracking analysis

EV samples were characterized and quantified using ZetaView (Particle Metrix GmbH, Germany) Nanoparticle Tracking Analysis (NTA), equipped with a sample cell and two lasers (488 nm and 640 nm). ZetaView 8.05.14_SP7 software was used. After calibration with 100 nm polystyrene beads, samples were diluted in 0.22-μm-filtered PBS 1X and injected using a 1-mL syringe. Size distribution analyses of 11 different positions were performed for each sample on at least three different sEV preparations. The particle number was then normalized to the initial number of seeded cells.

### 2.14 Non-conventional flow cytometry analysis of EVs

For flow cytometry characterization, EVs were suspended in 0.22-μm-filtered PBS/EDTA, distributed in flow cytometry tubes (1 × 10^8^ EVs/tube), and stained with 1 μM CFDA-SE (Vybrant^TM^ CFDA SE Cell Tracer Kit, Thermo Fisher Scientific) at RT to visualize intact vesicles. Since CFDA-SE diffuses within vesicles only at RT, a control at 4°C was also included. A mixture of fluorescent beads with a diameter ranging from 100 to 900 nm (Megamix-Plus FSC and Megamix-Plus SSC, Biocytex, France) was used to discriminate EV size. Expression of typical EV markers, such as CD9 (APC Mouse Anti-Human CD9, 312108, BioLegend, San Diego, CA, United States), CD63 (PE-CF594 Mouse Anti-Human CD63, 565403, BD Biosciences), and CD81 (BV421 Mouse Anti-Human CD81, 740079, BD Biosciences), was evaluated within the CFDA-SE positive events. All the evaluations were performed using a CytoFLEX flow cytometer (Beckman Coulter), and data were analyzed using FlowJo. Dimensional analysis was performed by creating three dimensional gates (EVs <100 nm, 100 nm < EVs <160 nm, and 160 nm < EVs <900 nm).

### 2.15 Transmission electron microscopy

The EV preparations from 2D cultures and from 3D spheroids of MSCs were re-suspended in 20 μL PBS (pH 7.4) and fixed by adding an equal volume of 2% paraformaldehyde in 0.1 mol/L phosphate buffer (pH 7.4). EVs were then adsorbed for 10 min onto formvar-carbon-coated copper grids by floating the grids on 4 μL drops on parafilm. Subsequently, grids with adhered vesicles were rinsed in PBS and negatively stained with aqueous 2% uranyl acetate for 5 min at room temperature. Stained grids were then embedded in ice-cold 2.5% methylcellulose for improved preservation and air-dried before examination. Electron micrographs were obtained using a Hitachi 120 kV transmission electron microscope (HT7800 series, Tokyo, Japan) equipped with a MegaView III digital camera and RADIUS 2.0 software (EMSIS, Muenster, Germany).

### 2.16 HUVEC culture

HUVECs (ATCC^®^ CRL-1730™) were plated on a T75-cm^2^ flask and cultured in F12-K medium (ATCC^®^), supplemented with 30 mg/mL of endothelial cell growth supplement (Sigma-Aldrich, St. Louis, MI, United States), 10% FBS, 100 U/mL of penicillin/streptomycin, and 0.1 mg/mL of heparin (Pharmatex) (complete medium). For the following test, HUVECs were used at passage number 5. For each test, positive and negative controls were carried out by incubating cells in complete and F12-K media, supplemented with 100 U/mL of penicillin/streptomycin (serum-free medium, SF medium), respectively. The effect of different fractions of the MSC-derived secretome on HUVEC proliferation, migration, and tube formation was evaluated.

### 2.17 HUVEC proliferation assay

HUVECs were seeded in a 96-well plate (10,000 cells/well) and cultured in the complete medium for 24 h to allow cell attachment. To synchronize the cell cycle, HUVECs were incubated with the SF medium for 2 h. HUVEC treatments were performed for 24 h using different secretome fractions (CM, DM, mEVs, and sEVs) derived from 30,000 MSCs. Cell proliferation was then measured using the BrdU proliferation assay, following the manufacturer’s instructions (Cell Proliferation ELISA, BrdU colorimetric, Roche, Basel, Switzerland). Data were normalized to a negative control.

### 2.18 HUVEC migration assay

To evaluate MSC-derived CM, DM, and EV ability to induce HUVEC migration, a Transwell system (Corning, New York, United States) was used. Transwells, with a pore size of 8 μm, were placed in a 24-well plate, and 50,000 cells re-suspended in the SF medium were seeded inside the inserts. Secretome fractions (CM, DM, mEVs, and sEVs) derived from 150,000 MSCs of the different culture conditions were put in the lower chamber. After 9 h of incubation at 37°C and 5% CO_2_, inserts were washed with PBS, and the non-migrated cells in the upper part of the membrane were gently removed using a cotton swab. Migrated cells were fixed in 10% buffered formalin for 10 min and stained with 1% methylene blue for 30 min. Images were acquired using a Leica DMi1 inverted phase microscope, and analysis was performed on at least five different areas using the ImageJ Cell Counter plugin. Data were normalized to a positive control.

### 2.19 Tube formation assay

Wells of a 96-well plate were coated with 50 μL of Matrigel (Corning) and incubated at 37°C for 30 min to allow Matrigel polymerization. Later, 20,000 HUVECs, re-suspended in the SF medium, were seeded on the top of the Matrigel and incubated at 37°C and 5% CO_2_ for 6 h in the presence of different secretome fractions (CM, DM, mEVs, and sEVs) derived from 60,000 MSCs. Images were acquired using a Leica DMi1 inverted phase microscope, and analysis was performed on at least three different areas using the ImageJ Angiogenesis Analyzer plugin (Carpentier et al., 2020). Data were normalized to a positive control.

### 2.20 Statistical analysis

Data were analyzed using GraphPad Prism 8.0 (GraphPad Software, Inc.), and they are shown as mean ± standard deviation (SD). Statistical analysis of differences between multiple groups was performed using a two-way or one-way ANOVA, followed by Tukey’s multiple comparison test. The level of significance was set at *p* < 0.05.

## 3 Results

### 3.1 MSCs in spheroid cultures maintain stemness characteristics but exhibit morphological alterations

Compared to conventional 2D culture, spheroids represent a promising alternative (Langan et al., 2016). On this basis, we evaluated whether spheroid size might influence the behavior and paracrine activity of human MSCs.

**FIGURE 1:**
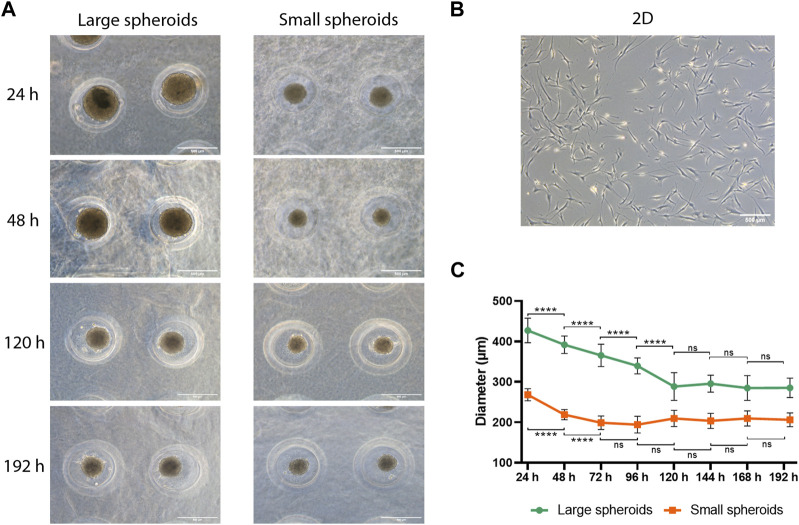
Spheroid dimensional analysis. **(A)** Representative images of large and small spheroids at different culture time points (24, 48, 120, and 192 h); scale bar = 500 µm. **(B)** Representative image of 2D MSC culture used as the control; scale bar = 500 µm. **(C)** Dimensional analysis of spheroid diameter variation along culture time. Diameter was calculated on 100 spheroids for each time point and replicate using ImageJ. Data are represented as the mean ± SD. **** *p*-value <0.0001 (N = 4, two-way ANOVA and Tukey’s multiple comparison).

Two different sizes of spheroids were generated using human MSCs: large (diameter ∼300 μm) and small spheroids (diameter ∼200 μm), which originated from approximately 2,600 and 1,000 cells, respectively (Figure 1A). 2D cultured MSC show a classical fibroblast like spindle-shaped cells morphology (Figure 1B). Spheroid diameter was measured over time, and interestingly, both large and small spheroids showed a significant size decrease during culture (Figure 1C; Supplementary Table S2). In addition, we highlighted size stabilization in small spheroids after 72 h of culture, while the diameter of large spheroids showed a constant decreasing trend until 120 h of culture, when the diameter became stable.

**FIGURE 2:**
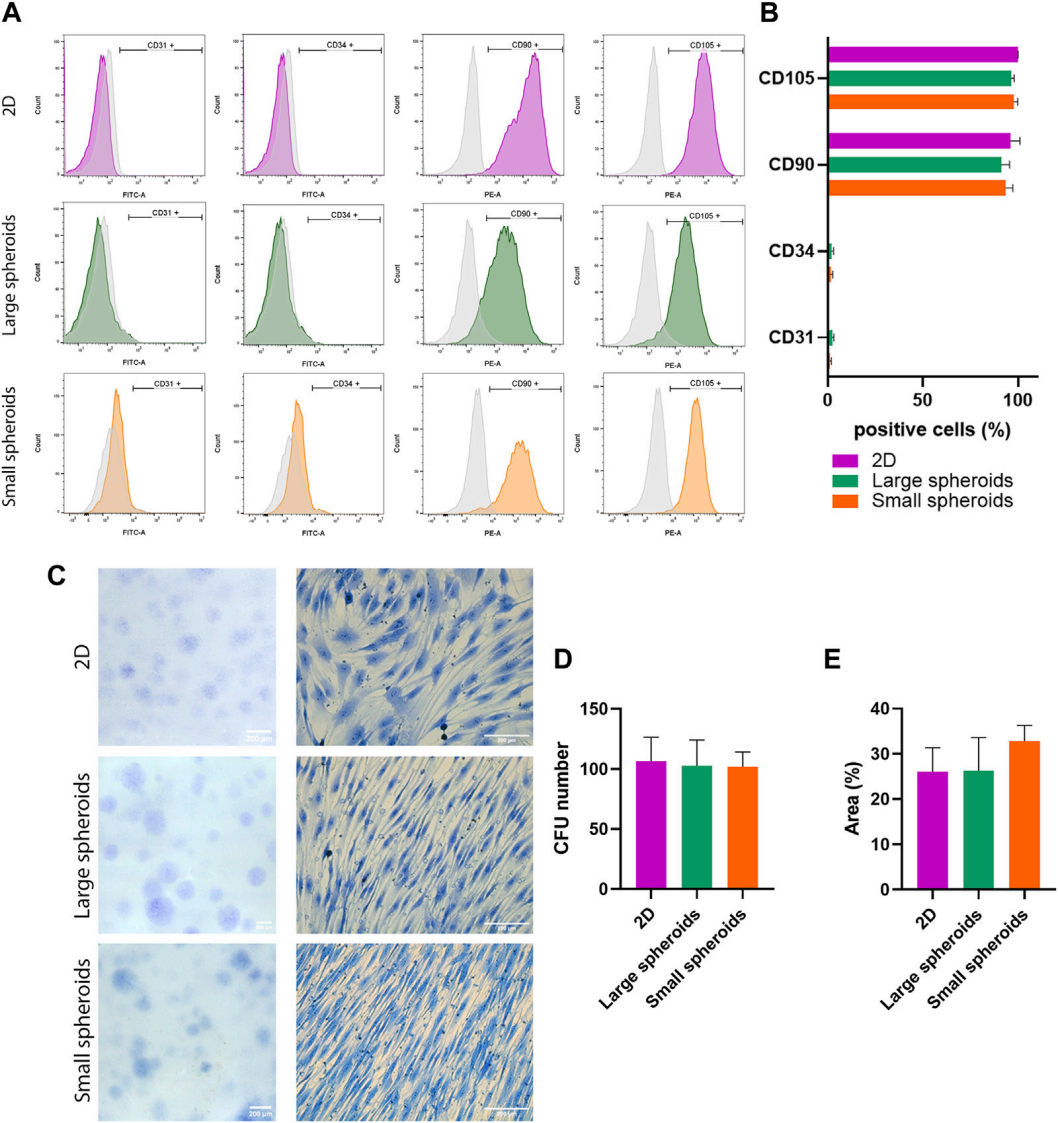
MSC phenotype and self-renewal ability evaluation. **(A)** Representative histograms reporting flow cytometry analysis of MSCs cultured in 2D (purple), large (green), or small (orange) spheroids. Histogram curves identify cells reacting with CD31-, CD34-, CD90-, and CD105-specific antibodies. Area under the gray curve identifies the reaction of the cells with the correspondent non-reactive immunoglobulin of the same isotype (isotype control). Data are representative of three independent experiments. **(B)** Flow cytometry analysis of MSCs cultured in 2D, large, or small spheroids. Histogram shows the average percentage of positive events for each marker. Data are represented as the mean ± SD (N = 3). **(C)** Representative images of CFU-f (left) and a magnification showing the colony cell morphology (right); scale bar = 200 µm. **(D)** Number of colonies and **(E)** area of the Petri dish occupied by colonies of MSCs derived from 2D, large, and small spheroid cultures. Data are represented as the mean ± SD (N = 3).

To verify that stemness properties were not affected by the culture method, surface phenotype, clonogenic potential, and trilineage differentiation potential (Supplementary Figure S1) were further investigated. The surface phenotype was evaluated using flow cytometry, assessing putative negative markers CD31 and CD34 and positive markers CD90 and CD105. No statistically significant alteration was observed between different experimental groups (Figures 2A, B), demonstrating that cells retain their typical phenotype independently from the culture condition.

The clonogenic potential, defined as the ability to form colonies starting from a single cell (CFU-f: colony-forming unit), was investigated. MSCs from 2D, large, and small spheroid culture conditions were seeded at low density, and their macro- and microscopic morphology was evaluated after 2 weeks. Microscopic morphology showed significant differences between different culture conditions (Figure 2C). MSC-derived colonies cultured in 2D showed fibroblast-like, spindle-shaped cell morphology with larger cells compared to spheroid-derived CFUs, which have a smaller size and elongated spindle-shaped morphology with a more compact organization. Interestingly, this peculiarity was enhanced in CFU derived from small spheroids. Furthermore, the total number of colonies was analyzed, and no significant difference was observed (Figure 2D). Finally, the surface area covered by colonies was analyzed (Figure 2E), and no significant difference was observed.

### 3.2 Spheroid size influences the establishment of a hypoxic core

**FIGURE 3:**
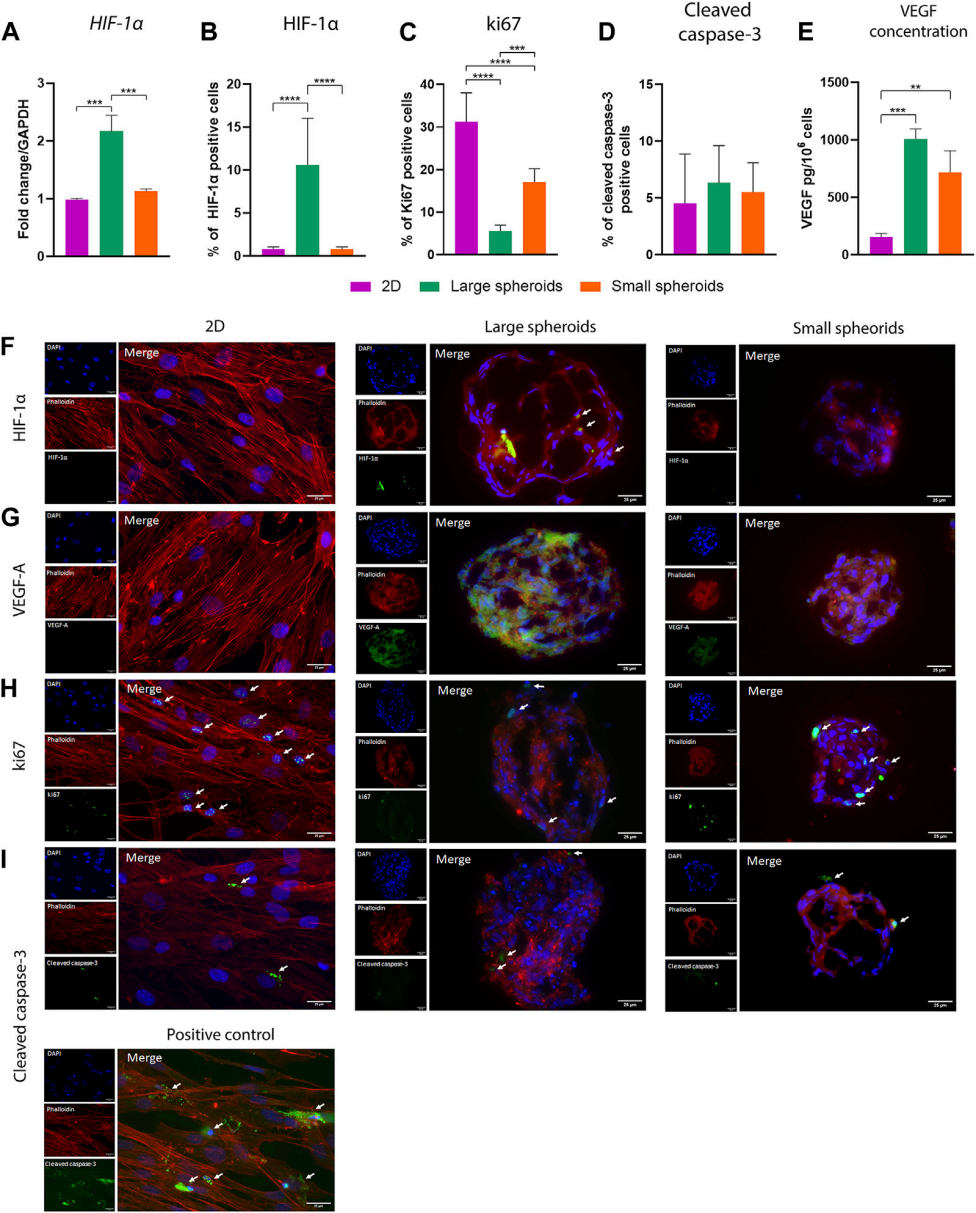
Spheroid size influences the generation of a hypoxic core. **(A)** Quantitative real-time PCR for the expression of *HIF-1α* (hypoxia-inducible factor alpha). *HIF-1α* expression of the 2D culture was compared to the expression in the 3D conditions. Data are represented as the mean ± SD. *** *p*-value <0.001 (N = 3, one-way ANOVA). Quantification of **(B)** HIF-1α-, **(C)** ki67-, and **(D)** cleaved caspase-3-positive cells on immunostaining. Positive cells have been counted considering 10 different regions of interest (ROI) for each replicate, with a mean diameter of 342.29 ± 20.90 µm for large spheroids and 194.43 ± 9.20 µm for small spheroids. Data are represented as the mean ± SD. *** *p*-value <0.001 and **** *p*-value <0.0001 (N = 3, one-way ANOVA). **(E)** Quantification of VEGF in the conditioned medium using the ELISA assay. Data are represented as the mean ± SD. ** *p*-value <0.01 and *** *p*-value <0.001 (N = 3, one-way ANOVA). Representative images of immunostaining for **(F)** HIF-1α, **(G)** VEGF-A, **(H)** ki67, and **(I)** cleaved caspase-3 on different culture conditions. For all the images from the top, pictures show the nuclei (DAPI), phalloidin, and the investigated markers mentioned before, along with a merged view; scale bar = 25 µm. In all the images, white arrows indicate positive cells.

Cell response to hypoxic stress is mainly mediated by hypoxia-inducible factors (HIFs). To study the influence of diameter on the onset of hypoxia in spheroids, mRNA expression levels of the transcriptional factor hypoxia-induced factor-1α (*HIF-1α*) were investigated using qRT-PCR (Figure 3A). *HIF-1α* was strongly overexpressed in large spheroids, compared to both 2D-cultured MSCs and small spheroids, where no differences were highlighted. To assess the localization of HIF-1α inside the 3D structure, its expression was further analyzed using immunofluorescence. Figure 3B shows the quantification of HIF-1α-positive cells, and Figure 3F shows a localization of HIF-1α in the central core of large spheroids, as highlighted by white arrows. No HIF-1α expression was found in small spheroids, suggesting that a small diameter may support the generation of a less strong gradient, allowing nutrients and oxygen to reach the central region.

Based on the different expression levels of *HIF-1α*, the expression of *VEGF-A*, a marker deeply interconnected with HIF-1α and hypoxia, was also investigated. VEGF secretion in the conditioned medium was also quantified using ELISA (Figure 3E). A significantly higher concentration of VEGF was observed in both large and small spheroids compared to 2D-derived CM, suggesting that spheroid culture can stimulate a higher secretion of VEGF compared to the classical monolayer culture. Expression of VEGF-A was also investigated via immunofluorescence staining (Figure 3G), which highlighted a strong signal in the internal regions of large spheroids. On the contrary, a weak but still detectable signal was observed in small spheroids and totally absent in 2D cultures. All these data together suggest that VEGF-A secretion is partially dependent on the culture condition.

In addition, the expression of the proliferative marker, ki67, was also investigated (Figures 3C, H). A decreased expression was found in large spheroids, while enhanced ki67 expression was observed in small spheroids, particularly in the external regions (white arrows in Figure 3H). However, both large and small spheroids exhibited lower ki67 expression compared to the 2D cultured control, suggesting a reduced proliferative capability in 3D culture compared to monolayer culture.

On the other hand, the expression of cleaved caspase-3 was also investigated (Figures 3D, I) to assess whether 3D culture influences levels of apoptosis. Staining specificity was confirmed by treating MSCs for 72 h with 10 μM etoposide, a molecule that can induce apoptosis, as a positive control. Interestingly, similar levels of apoptosis were observed between the different conditions, suggesting that this alternative culture method does not enhance the apoptotic process. Cell death was also assessed via the LIVE/DEAD assay (Supplementary Figure S2), showing that no PI-positive cells are present in the structure.

### 3.3 Spheroid culture influences cellular senescence in MSCs

**FIGURE 4:**
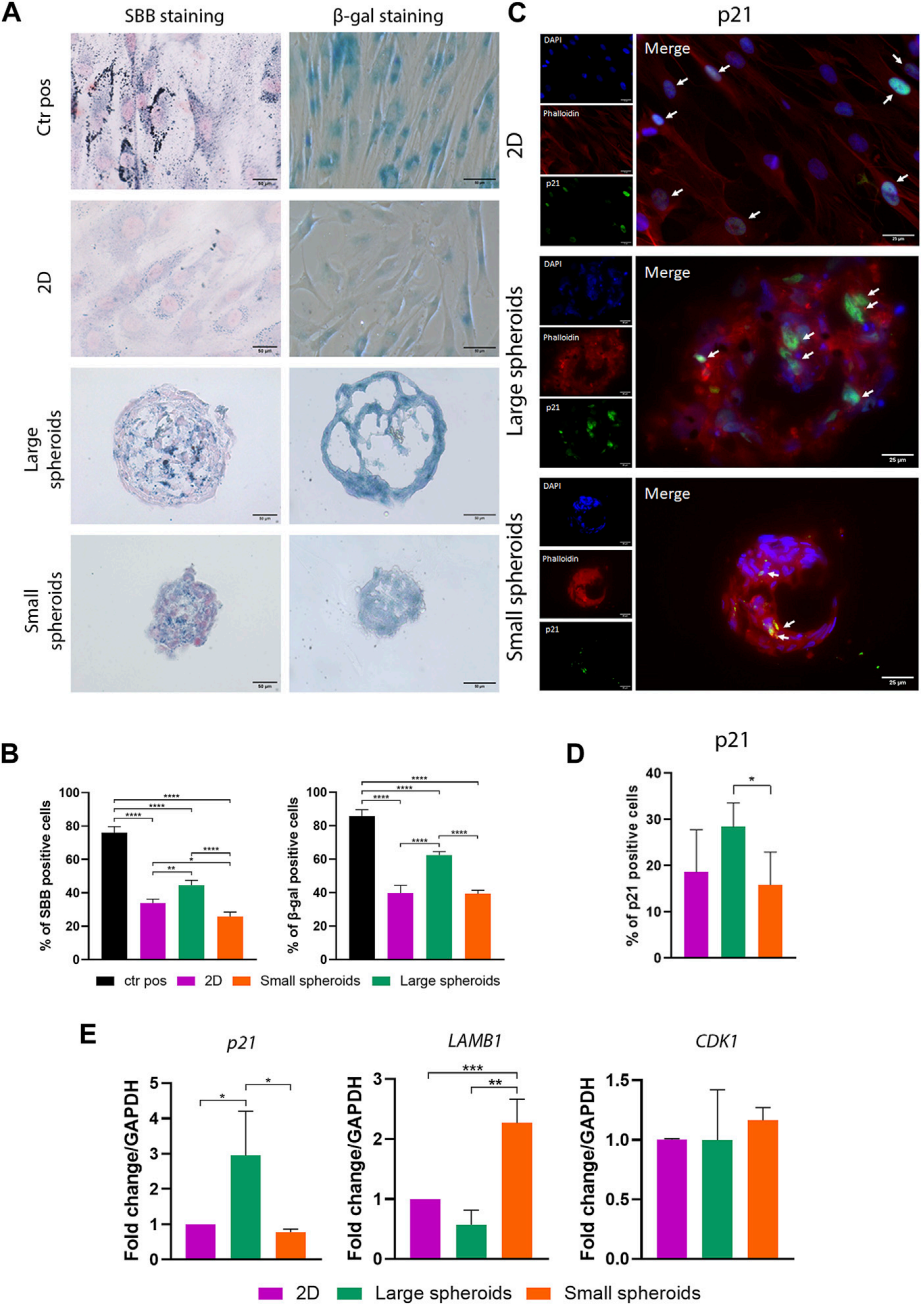
Spheroid culture influences cellular senescence in MSCs. **(A)** Representative images of Sudan black B and β-galactosidase staining of the different culture conditions; scale bar = 50 µm. Ctr pos, positive control. **(B)** Quantification of SBB- and β-galactosidase-positive cells. SBB- and β-galactosidase-positive cells have been counted considering 10 different regions of interest for each replicate with a mean diameter of 338.14 ± 19.62 µm for large spheroids and 187.43 ± 13.26 µm for small spheroids. Data are represented as the mean ± SD. * *p*-value <0.05, ** *p*-value <0.01, and **** *p*-value <0.0001 (N = 4, one-way ANOVA). **(C)** Representative images of immunostaining for p21 on different culture conditions. From the top, pictures show the nuclei (DAPI), phalloidin, p21, and their merge view; scale bar = 25 µm. **(D)** Quantification of p21-positive cells on immunostaining. Positive cells have been counted considering 10 different regions of interest for each replicate, with a mean diameter of 332.12 ± 18.40 µm for large spheroids and 193.35 ± 7.22 µm for small spheroids. **(E)** Quantitative real-time PCR for the expression of *p21*, *LAMB1* (lamin subunit beta 1), and *CDK1* (cyclin-dependent kinase 1); data are represented as the mean ± SD. * *p*-value <0.05, ** *p*-value <0.01, and *** *p*-value <0.001 (N = 3, one-way ANOVA).

Culture conditions may have an important influence on cellular senescence onset. Senescence was investigated by SBB staining, β-galactosidase activity, and qRT-PCR on senescence-related genes. SBB is a dye used to stain lipofuscin, a lysosomal product accumulated inside the cells following lysosomal dysfunction, usually occurring in senescent cells (Figure 4A). A positive control (Ctr pos), obtained by treating MSCs with 20 μM H_2_O_2_, was also carried out to confirm dye specificity. Quantitative analysis (Figure 4B) showed that SBB-positive cell number significantly increased in large spheroids compared to 2D cultures, suggesting that spheroid size may influence cellular senescence. On the other hand, levels of cellular senescence are decreased in small spheroids compared to both 2D and large spheroid cultures.

β-Galactosidase is an enzyme usually expressed by senescent cells. The evaluation of β-galactosidase activity is one of the gold standard methods used to evaluate the presence of senescent cells. Positive control, as also observed from SBB staining, shows the presence of a high percentage of senescent cells (Figure 4A), confirming staining specificity. Quantitative analysis (Figure 4B) revealed similar results to SBB staining. A higher number of β-galactosidase-positive cells were identified in large spheroids compared to 2D, while a comparable number of β-galactosidase-positive cells were found in small spheroids and the 2D condition.

Similar results were obtained by analyzing the expression of p21 using immunostaining (Figures 4C, D), a protein usually upregulated in senescent cells, where it inhibits the formation of the CDK2–cyclin E complex involved in the G1-S cell cycle checkpoint (González-Gualda et al., 2021; Schmitt et al., 2022). A higher number of p21-positive cells were observed in large spheroids compared to the 2D culture, while a similar number of p21-positive cells were found in small spheroids and the 2D culture. In addition, we highlighted that a percentage of senescent cells can also be detected in the 2D culture in all the performed analyses for SBB, β-galactosidase, and p21 staining (Figures 4A–D). This may be due to the old age (average 75 years) of MSC donors, which could explain the co-isolation of a senescent fraction of cells (Gnani et al., 2019; Kaminska et al., 2021).

In addition, cellular senescence was further investigated by assessing mRNA levels of genes normally dysregulated during the senescent process, such as *p21*, *lamin subunit B1* (*LAMB1*), and *cyclin-dependent kinase-1* (*CDK1*), using real-time PCR (Figure 4E). mRNA levels of *p21* were upregulated in large spheroids compared to both 2D culture and small spheroids, where similar expression levels of *p21* were found. *LAMB1*, a key component of the nuclear lamina, is another key hallmark of cellular senescence since it is usually downregulated in senescent cells, leading to the loss of nuclear integrity and DNA leakage in the cytoplasm (González-Gualda et al., 2021; Kumari and Jat, 2021). A slight, not-significant downregulation of this marker was observed in large spheroids compared to the 2D culture. Intriguingly, small spheroids showed a strong upregulation of *LAMB1* compared to both 2D culture and large spheroids. Finally, no significant variations were observed in the expression of *CDK-1*.

### 3.4 MSC spheroid-derived secretome showed upregulation of pro-angiogenic and inflammatory cytokines

**FIGURE 5:**
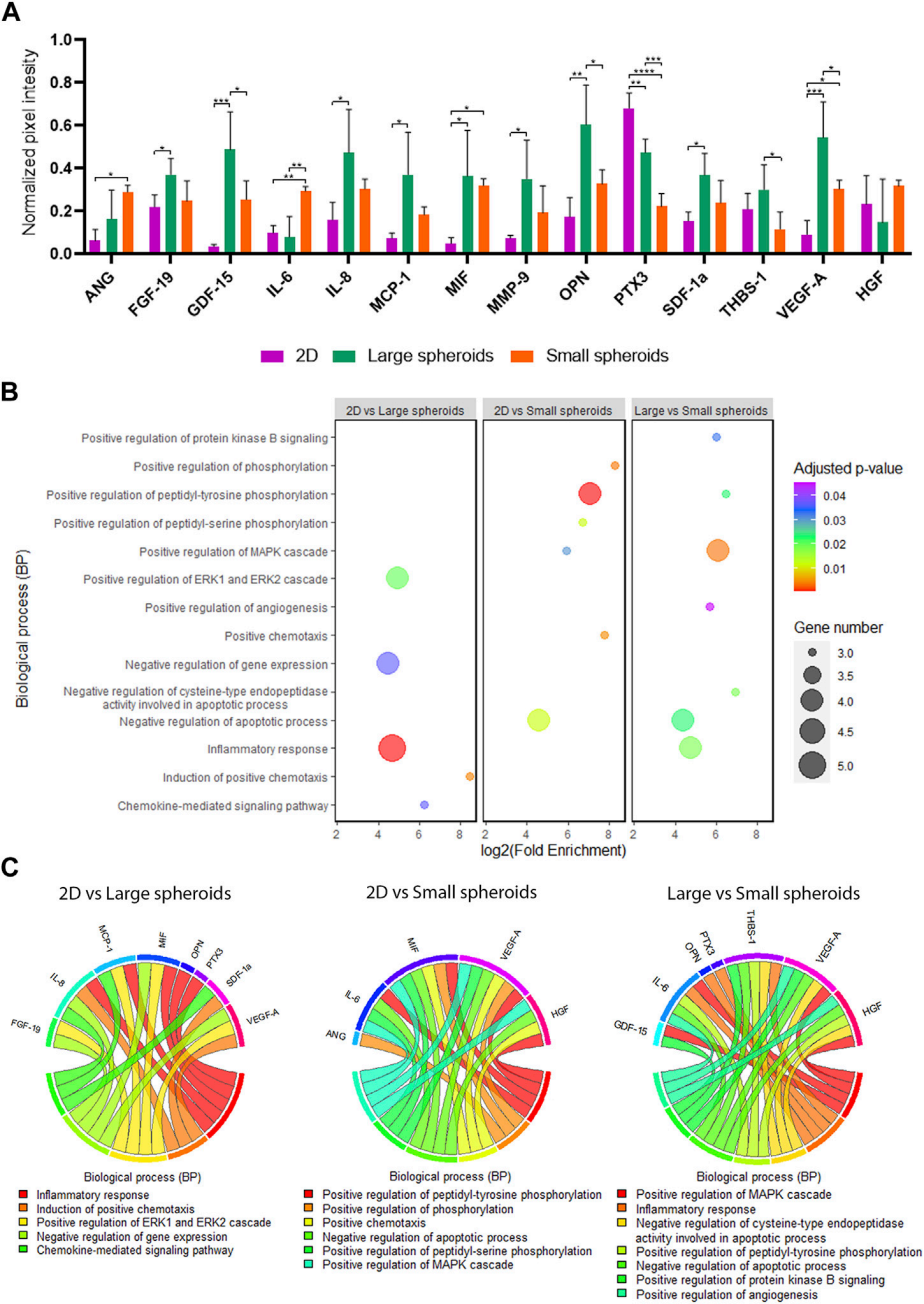
Secretome profiling. **(A)** Quantification of normalized pixel intensity for each modulated protein on cytokine arrays of CM derived from 2D, large, and small spheroids MSC culture. Data are represented as the mean ± SD; * *p*-value <0.05, ** *p*-value <0.01, *** *p*-value <0.001, and **** *p*-value <0.0001 (N = 4, one-way ANOVA). ANG, angiogenin; FGF-19, fibroblast growth factor 19; GDF-15, growth differentiation factor 15; IL-6, interleukin 6; IL-8, interleukin 8; MCP-1, monocyte chemoattractant protein 1; MIF, macrophage migration inhibitory factor; MMP-9, matrix metallopeptidase 9; OPN, osteopontin; PTX3, pentraxin 3; SDF1a, stromal cell-derived factor 1; THBS-1: thrombospondin 1; VEGF-A, vascular endothelial growth factor-A; HGF, hepatocyte growth factor. **(B)** Bubble plot of Gene Ontology analysis of the BP term on modulated cytokines identified with cytokine arrays. **(C)** Chord plot showing the cytokine involvement in the different biological processes identified with Gene Ontology analysis.

In order to evaluate the secretome composition, CM were analyzed using the XL Proteome Profiler to evaluate differently secreted molecules between the three culture conditions, and important differences were highlighted (Figure 5A; Supplementary Figure S3). Compared to 2D, large spheroid-derived secretome showed upregulation of different cytokines, such as fibroblast growth factor-19 (FGF-19), growth differentiation factor-15 (GDF-15), interleukin-8 (IL-8), monocyte chemoattractant protein-1 (MCP-1), macrophage migration inhibitory factor (MIF), matrix metallopeptidase-9 (MMP-9), osteopontin (OPN), stromal derived factor-1-alpha (SDF-1α), and VEGF-A, and the downregulation of pentraxin-3 (PTX3).

In addition, compared to small spheroid-derived secretome, large-spheroid-derived secretome showed upregulation of GDF-15, OPN, PTX3, THBS-1, and VEGF-A and downregulation of interleukin-6 (IL-6) and HGF (*p* = 0.08).

On the other hand, compared to 2D, small spheroid-derived secretome exhibited an upregulation of ANG, IL-6, VEGF-A, and MIF and a downregulation of PTX3.

Furthermore, as reported in Supplementary Figure S4, the different biological replicates analyzed have similar behavior underlying data consistency. In addition, the dendrogram located on top of the heatmap highlights that all the biological replicates derived from a specific condition are clustered in the same branch.

In Figure 5B, the main biological processes identified by GO analysis in different comparisons are presented, and in Figure 5C, the main factors involved in these different biological processes are shown.

### 3.5 MSC spheroids secrete a higher quantity of EVs compared to 2D

**FIGURE 6:**
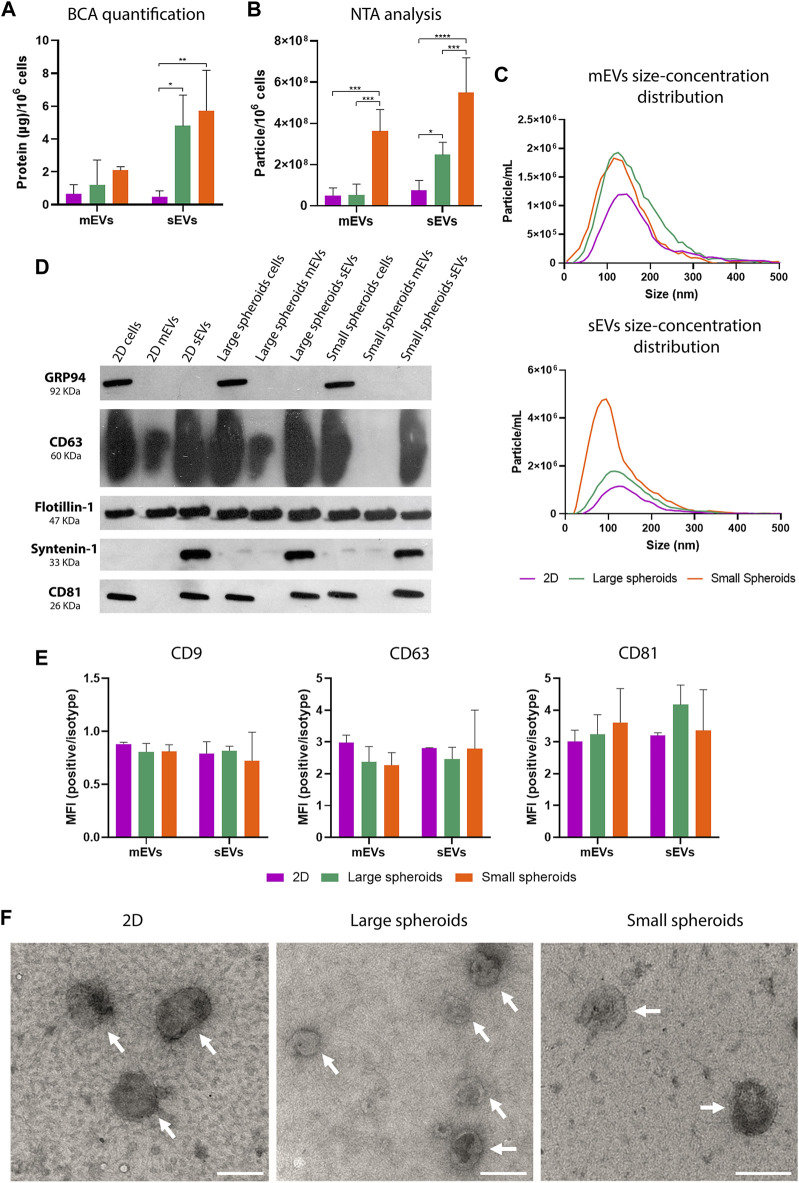
MSC spheroids secrete higher quantity of EVs compared to 2D. **(A)** Quantification of protein content on mEVs and sEVs using the BCA assay; data are normalized to the cell number (µg/10^6^ cells). Data are represented as the mean ± SD; * *p*-value <0.05 and ** *p*-value <0.01 (N = 4, two-way ANOVA and Tukey’s multiple comparison). **(B)** NTA of particle concentration, expressed as particle/10^6^ cells. Data are represented as the mean ± SD; * *p*-value <0.05, *** *p*-value <0.001, and **** *p*-value <0.0001 (N = 4, two-way ANOVA and Tukey’s multiple comparison). **(C)** Representative NTA size distribution analysis of mEVs and sEVs (N = 4). **(D)** Western blot analysis on 2D, large, and small spheroid MSC culture-derived mEVs and sEVs; cell lysates were also loaded as controls. Specific expression of CD63, CD81, flotillin-1, syntenin-1, and GRP94 was investigated. **(E)** Quantification on flow cytometry analysis of CD9-, CD63-, and CD81-positive events in the CFDA-SE gate. Data are shown as ratio between mean fluorescence intensity (MFI) of EVs stained with specific antibody and the correspondent isotype control (relative MFI) (N = 3). **(F)** TEM micrographs of isolated mEVs and sEVs. Scale bar = 200 nm. White arrows indicate EVs.

EVs were isolated from the conditioned medium (CM) obtained by maintaining MSCs in the SF medium for 24 h at 37°C and 5% CO_2_. The BCA assay revealed a higher protein concentration in sEVs derived from large and small spheroids, compared to 2D-derived sEVs, with no significant difference between large and small spheroid-derived sEVs (Figure 6A). No significant difference was highlighted in mEV protein concentration.

NTA of mEVs and sEVs (Figures 6B, C) showed an increased concentration of mEVs derived from small spheroids and a higher concentration of sEVs derived both from large and small spheroid MSCs compared to 2D. Furthermore, a higher concentration of sEVs was also observed in small spheroids compared to large spheroids. NTA of EV size showed a consistent reduction of dimension in sEVs derived from all conditions (Table 2) with no significant alteration between different culture conditions.

**TABLE 2:** NTA of size of mEVs and sEVs isolated from 2D, large, and small spheroids. Data are reported as the mean ± SD (N = 4). mEVs: medium-sized EVs, sEVs: small-sized EVs.

	Mean size	Peak size
**2D mEVs**	155.18 ± 17.01 nm	150.40 ± 23.68 nm
**2D sEVs**	132.50 ± 3.68 nm	119.55 ± 9.41 nm
**Large spheroid mEVs**	159.13 ± 26.08 nm	150.65 ± 38.22 nm
**Large spheroid sEVs**	134.83 ± 11.61 nm	118.15 ± 17.94 nm
**Small spheroid mEVs**	153.10 ± 13.71 nm	148.05 ± 20.51 nm
**Small spheroid sEVs**	136.10 ± 6.53 nm	117.43 ± 4.78 nm

Western blot analysis of mEVs, sEVs, and cell lysate (Figure 6D) was used to investigate the expression of classical vesicular markers (flotillin-1, syntenin-1, CD63, and CD81). As expected, the negative control GRP94, a chaperon needed to assist the folding and assembly of secreted and membrane proteins in ER (Eletto et al., 2010), was expressed only in cell lysate. Syntenin-1, a classical marker of sEVs, was expressed only in this population in all the culture conditions. Flotillin-1 was expressed in both EV populations and cell lysate. Interestingly, the expression of tetraspanins CD63 and CD81 was detected in sEVs derived from all culture conditions. CD81 was not observed in any population of mEVs, while CD63 was poorly observable only in mEVs derived from 2D and large spheroids and not observed in mEVs derived from small spheroids.

EVs were also analyzed using a non-conventional flow cytometry approach to evaluate the expression of tetraspanins (CD9, CD63, and CD81). This approach is based on the use of fluorescent dimensional beads of known diameter (Supplementary Figure S6A) and the lipophilic tracer, CFDA-SE, that passively diffuses inside EVs and interacts with intraventricular enzymes at RT (Supplementary Figure S6B). An appropriate dimensional gate was designed using EVs stained with CFDA-SE at 4°C, and the expression levels of CD9, CD63, and CD81 were assessed in the CFDA-SE-positive events. As reported in Figure 6E, CD9 is the least expressed marker, while expression levels of CD63 and CD81 were similar in different culture conditions. Moreover, similar levels of tetraspanin expression were observed between mEVs and sEVs. Dimensional analysis was performed by creating three different gates (EVs<100 nm, 100 nm<EVs<160 nm, and 160 nm<EVs<900 nm). As shown in Table 3 and Supplementary Figure S6D, an enrichment of the EVs <100 nm was observed in all the conditions, particularly in sEVs. Similar levels of EVs in the gate 100 nm<EVs<160 nm were present in the different EV preparations, and a small percentage of EVs with dimensions higher than 160 nm was observed. EVs were further characterized by transmission electron microscopy (Figure 6F). TEM micrographs confirmed that all the EV preparations are enriched in intact vesicles surrounded by a lipid bilayer (white arrows in Figure 6F).

**TABLE 3:** Percentage of CDFA-SE-positive events in the considered three-dimensional gates (EVs <100 nm, 100 nm<EVs<160 nm, and 160 nm<EVs<900 nm). Data are reported as the mean ± SD (N = 3). mEVs: medium-sized EVs, sEVs: small-sized EVs.

	EVs <100 nm	100 nm<EVs<160 nm	160 nm<EVs<900 nm
**2D mEVs**	60.70% ± 18.53%	15.57% ± 3.37%	14.60% ± 8.80%
**2D sEVs**	68.33% ± 13.09%	9.58% ± 5.85%	16.13% ± 10.56%
**Large spheroid mEVs**	56.77% ± 1.70%	27.53% ± 0.38%	9.56% ± 1.67%
**Large spheroid sEVs**	65.40% ± 5.75%	11.73% ± 1.04%	20.50% ± 6.55%
**Small spheroid mEVs**	50.67% ± 6.04%	28.93% ± 10.27%	12.46% ± 5.01%
**Small spheroid sEVs**	63.20% ± 6.41%	17.23% ± 3.72%	11.34% ± 3.75%

### 3.6 Spheroid culture increased the angiogenic potential of MSC-derived soluble factors and EVs

**FIGURE 7:**
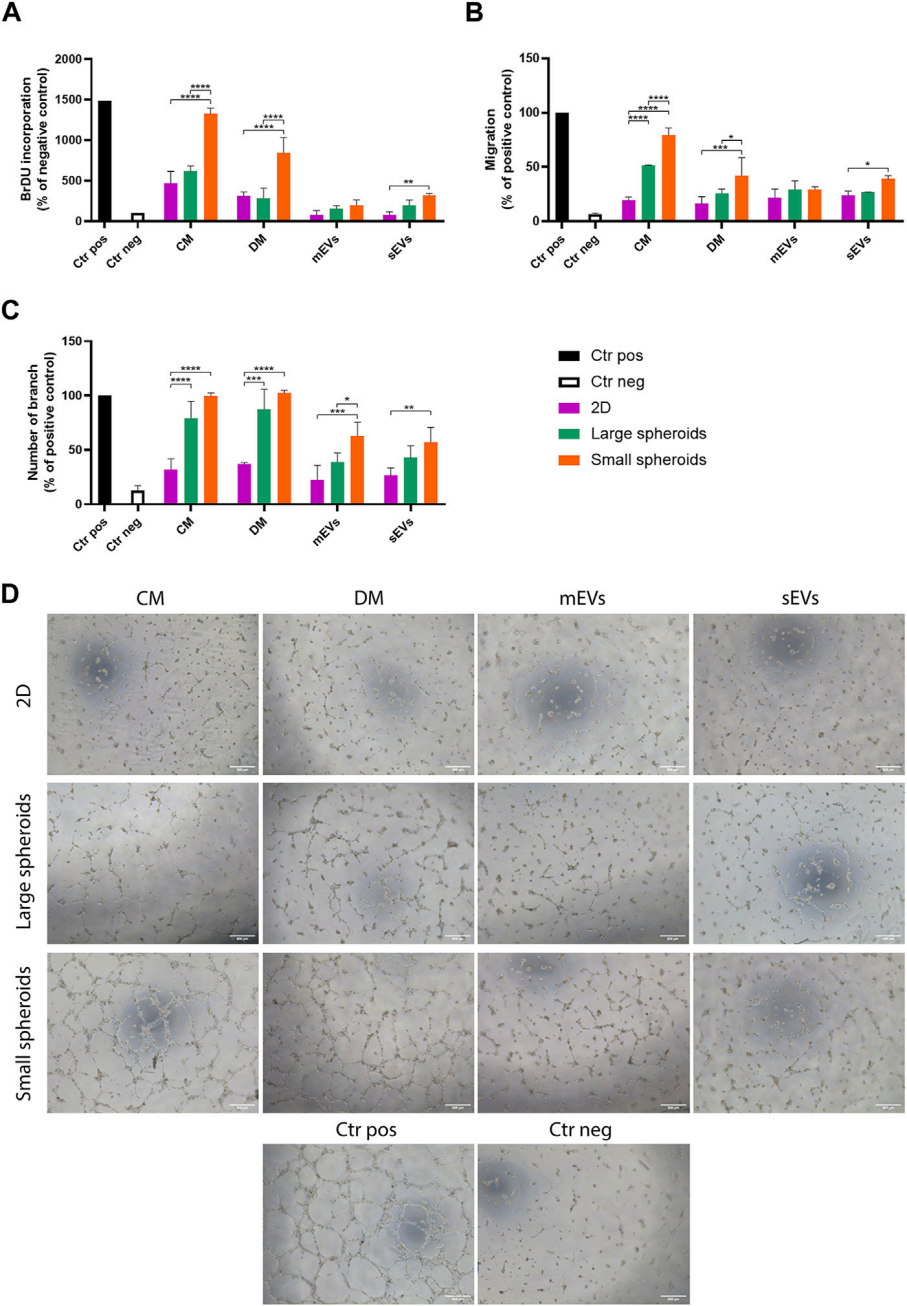
Effects of MSC secretome fractions on HUVEC proliferation, migration, and tube formation. **(A)** HUVEC proliferation was assessed via the BrdU incorporation assay; **(B)** migration was assessed via the Transwell migration assay. **(C)** Quantitative analysis of number of branches in the tube formation assay. Data are represented as the mean ± SD. * *p*-value <0.05, ** *p*-value <0.01, *** *p*-value <0.001, and **** *p*-value <0.0001 (N = 3, two-way ANOVA and Tukey’s multiple comparison). Ctr pos, positive control; Ctr neg, negative control; CM, conditioned medium; DM, depleted medium; mEVs, medium-sized EVs; sEVs, small-sized EVs. **(D)** Representative images of the HUVEC tube formation assay; scale bar = 500 µm.

The angiogenic potential of the different secretome fractions (CM, DM, mEVs, and sEVs) derived from 2D, large, and small spheroids was evaluated on HUVECs by assessing their proliferation, migration, and ability to create tubes. Cell treatments were performed using a known amount of the different secretome fractions based on the cell number, and HUVECs were treated in all tests, maintaining a cell ratio of 1:3 (HUVEC:MSC). First, the effect of the different secretome fractions on HUVEC proliferation was investigated (Figure 7A). Interestingly, only small spheroid-derived secretome showed a strong effect on proliferation induction. More deeply, both CM and DM were able to induce significantly higher HUVEC proliferation compared to 2D and large spheroids. In addition, small spheroid-derived sEVs also showed a proliferative effect significantly more robust compared to 2D-derived sEVs, suggesting a synergistic effect influenced by both vesicular and soluble components. On the contrary, mEVs derived from all culture conditions did not show any effect on HUVEC proliferation.

Endothelial cells are able to migrate toward chemoattractant signals. A Transwell system was used to evaluate the migratory ability of HUVECs by inserting the different secretome fractions in the lower chamber and the cells in the upper chamber. Small spheroid-derived CM showed a stronger effect on HUVEC migration compared to large spheroid-derived CM, while the 2D-derived CM had low activity (Figure 7B). Small spheroid-derived DM showed a reduced effect compared to the CM. As observed before, small spheroid-derived sEVs induced a higher migratory effect on HUVECs compared to sEVs derived from the other conditions.

A tube formation assay was used to evaluate this specific aspect of the angiogenic process that starts only in the later stages, when cells migrate in the region of interest, proliferate to increase their number, and then start to create vessels (Adair and Montani, 2010). Different from the other tests, similar effects of CM and DM derived from the two spheroid cultures were observed (Figures 7C, D). In this assay, a significantly higher effect of both small spheroids-derived mEVs and sEVs was also observed.

Collectively, these results indicated that a possible synergic action of soluble factors and EVs can enhance the angiogenic potential of the small spheroid-derived secretome.

## 4 Discussion

The role of MSC-derived soluble factors and EVs in angiogenesis is crucial for the development of new therapeutic approaches, which may be used for the treatment of vasculogenesis-related disorders. In this study, we investigated how a different culture system could affect MSC properties and their angiogenic potential without the use of external priming such as hypoxia or cytokine treatments. Although these approaches are often successfully used to enhance specific MSC therapeutic potential, they present some limitations, including induction of immunogenicity, high costs, MSC source-related variability, and a lack of standardized protocols (Noronha et al., 2019; Noronha et al., 2021).

In this study, MSCs were cultured as spheroids of different sizes. Cellular senescence, secretome composition, EV secretion, and angiogenic potential were investigated to evaluate whether these aspects could be affected by the use of spheroid culture and the size of the spheroid.

First, the culture conditions do not affect the stemness of MSCs, which maintains their superficial phenotype and clonogenic potential, while cell morphology was influenced in terms of size reduction during culture time, which may be due to a cytoskeleton reorganization (Tsai et al., 2015; Cesarz and Tamama, 2016).

The cell morphology difference was mirrored in CFU-forming MSCs, where cells derived from 2D standard conditions showed enlarged morphology, in contrast to 3D conditions, especially in small spheroids, where smaller and extremely elongated cells were found. MSCs usually possess a classical fibroblastic morphology with spindle-shaped cells, which are lost when senescence occurs (Turinetto et al., 2016). As observed in our data, MSCs forming CFU under the 3D condition show a change in morphology compared to those under the 2D condition. This morphology alteration has already been described as a normally occurring event in MSC-cultured spheroids. The size of MSCs in spheroids is drastically smaller than cells in the 2D monolayer, resulting in a 75% reduction in individual cell volume (Cesarz and Tamama, 2016). It has been reported that the process of spheroid formation is characterized by an active cell rearrangement, where MSCs first form a loose network, followed by a gradual cell condensation over a central core. Spheroids compress progressively in the first few days due to a reduction in the amount of cytoplasm and cell volume (Bartosh et al., 2010).

The process of spheroid formation is responsible for the creation of a peculiar architecture, which generates a heterogeneous microenvironment characterized by gradients of different soluble molecules, including gases and nutrients, more accurately mimicking the *in vivo* milieu (Sart et al., 2014). Among these molecules, oxygen has been considered a key factor in regulating cell functions, including cell proliferation (Cheema et al., 2008). In more detail, an oxygen gradient is formed in the spheroids, with (i) a surface with a high concentration of oxygen, populated by cells with a high proliferation rate, (ii) a middle zone with a low concentration of oxygen, characterized by viable quiescent cells, and (iii) a hypoxic core in the central area, with necrotic and apoptotic cells. Oxygen availability is also known to regulate HIFs, which orchestrate the expression of hypoxia-associated genes involved in the regulation of stem cell properties, development, and metabolism (Grayson et al., 2007). We here demonstrated that HIF-1α is downregulated in small spheroids compared to large spheroids, suggesting that nutrients can diffuse more efficiently and reach the core. Therefore, the use of small spheroids could overcome this critical constraint, preventing this gradient formation (Mueller-Klieser, 1984).

The specific 3D organization also influences the diffusion of metabolic wastes, growth factors, and cytokines produced by MSC that might be translated into their increased internal levels (Sart et al., 2014). This could explain the upregulated expression of VEGF-A in large spheroids compared to small spheroids. Furthermore, substantial evidence has also reported that the hypoxic environment may be responsible for the upregulation of some growth factors, such as VEGF, FGF-2, and HGF (Bhang et al., 2011).

The effects of spheroid size on cell proliferation and apoptosis were also investigated. Interestingly, apoptotic levels were not enhanced in both spheroid conditions compared to the 2D culture. As reported in the literature, the short-term culture used in this study (96 h) and the dimension of the spheroids may not be sufficient to enhance the apoptotic process (Jauković et al., 2020). On the other hand, we observed that ki67 was strongly downregulated in large spheroids compared to small spheroids, suggesting impairment in cell proliferation. The literature indicates that less than 1% of 3D-cultured MSCs are actively dividing, with 90% arrested in the G0/G1 phase of the cell cycle (Bartosh et al., 2010). In addition, the block of cell proliferation in large spheroids may be related to different signals coming from suffering and senescent cells in the inner region that became stressed due to the lack of nutrients and oxygen, arresting the proliferative ability of more external cells.

Senescence is usually correlated with metabolic dysfunctions, leading to an alteration in MSC secretory ability, characterized by higher secretion of pro-inflammatory molecules capable of stimulating cellular senescence in neighboring cells (Marques et al., 2017). The presence of a higher number of senescent cells in large spheroids was confirmed by an increased activity of β-galactosidase (Hernandez-Segura et al., 2018) and accumulation of lipofuscin, linked with lysosomal dysfunction (Kumari and Jat, 2021).

Low proliferation levels in large spheroids are in accordance with the overexpression of *p21* observed, a protein involved in cell cycle arrest and usually upregulated at the onset of replicative senescence (González-Gualda et al., 2021). In addition, we observed a downregulation of *LAMB1*, a protein needed to ensure nuclear integrity and avoid the leakage of DNA in the cytosol, which can enhance cellular damage and fuel SASP secretion (Kumari and Jat, 2021). These findings confirmed that the establishment of a hypoxic region in the spheroids could increase cell damage and lead to the onset of replicative senescence.

Conversely, the absence of a strong hypoxic core in small spheroids is accompanied by decreased cellular senescence, as evidenced by reduced β-galactosidase activity and lipofuscin accumulation, in addition to *p21* downregulation. The upregulation of LAMB1 in small spheroids, usually downregulated in senescent cells (Freund et al., 2012), may minimize DNA loss in the cytoplasm, where it can fuel SASP secretion and enhance cellular damage.

Metabolic and redox alterations were also recently linked to the onset of replicative senescence. From the observed data, one hypothesis could be that in large spheroids, the hypoxic core led to an increase in ROS production, thereby stimulating the onset of replicative senescence (Yuan et al., 2020; Jeske et al., 2021). Given the significant difference highlighted between the two spheroid cultures, we suggest the adoption of small spheroids for the maintenance of MSCs, capable of reducing the onset of cellular senescence.

Different levels of cellular senescence are also mirrored by secretome composition, which is evaluated using an XL Proteome Profiler (Fernandez-Rebollo et al., 2020). Despite the limitation of the low number of cytokines analyzed in this method, we have observed an upregulation of cytokines known to be part of the SASPs (Basisty et al., 2020; Birch and Gil, 2020; Kumari and Jat, 2021) in large spheroids, such as GDF-15, IL-8, MCP-1, MMP-9, and THBS-1. As observed from GO analysis, the activation of the specific pathways ERK1 and ERK2, normally regulating cellular proliferation, might also be involved in the premature onset of cellular senescence in our large spheroid model (Zou et al., 2019). On the contrary, small spheroid-derived secretome exhibited expression of these cytokines comparable to 2D-cultured MSCs, confirming the hypothesis that the establishment of a hypoxic core is detrimental in terms of cellular senescence induction, while size reduction may help overcome this issue. As a confirmation of this hypothesis, GO analysis on large spheroids showed the presence of terms such as “inflammatory response,” confirming the upregulation of cytokines with pro-inflammatory activity, including the SASP factors previously cited, similarly downregulated under 2D and small spheroid conditions.

Remarkably, both 3D secretomes showed upregulation of MIF, a cytokine with proved rejuvenating effects on aged MSCs (Xia et al., 2015), and positive effects on cell proliferation and survival (Y. Zhang et al., 2019). The effect on cell proliferation was indeed detected only in small spheroids, where ki67 expression was maintained, contrary to large spheroids. This divergent cell response may be due to the high level of senescence-related cytokines, cell stress, and damage in the inner region of large spheroids. This hypothesis was confirmed by GO analysis, in which BPs related to negative regulation of apoptosis and proliferation, such as the MAPK pathway and protein kinase B signaling, were identified only in small spheroid-derived secretome.

Moreover, cytokine arrays performed on large spheroid secretome also revealed that large spheroid secretome is enriched in molecules showing pro-angiogenic, anti-angiogenic, and pro-inflammatory activity. More specifically, the identified cytokines included IL-8, known for its role in the process of tube formation in angiogenesis (Li et al., 2003) and possessing strong pro-inflammatory activity; MMP-9, a protease involved in ECM degradation (Almalki and Agrawal, 2016); SDF-1α, capable of stimulating HUVEC migration and tube formation (M. Zhang et al., 2017); and VEGF-A. On the other hand, a subset of anti-angiogenic cytokines was identified, including MCP-1, capable of inducing endothelial cell apoptosis (X. Zhang et al., 2011); PTX-3, an inflammation-related long pentraxin that blocks the angiogenic process by inhibiting FGFR activation (O’Neill et al., 2016); and THBS-1, a protein involved in the regulation of inflammatory process, also known as an endogenous inhibitor of angiogenesis, affecting endothelial cell proliferation, migration, and stimulating apoptosis (Lawler and Jack, 2012; Feng et al., 2015). GO analysis showed that the BP “inflammatory response” is activated by some cytokines with anti-angiogenic activity and is also able to induce stress in spheroid-cultured MSCs and stimulate the onset of cellular senescence.

On the contrary, small spheroid-derived secretome showed an upregulation of potent pro-angiogenic molecules, such as ANG, IL-6, able to stimulate HUVEC proliferation and migration (Fan et al., 2008), VEGF-A, and HGF, a cytokine with pro-angiogenic and anti-inflammatory properties (Rong et al., 2018). As also observed in GO analysis, terms related to “positive regulation of angiogenesis” as well as “negative regulation of apoptosis” (activated by molecules with strong pro-angiogenic activity such as HGF, VEGF-A, and IL-6) were found, suggesting the involvement of these cytokines in protecting endothelial cells from apoptosis. In addition, this culture condition also evidenced a downregulation of all the anti-angiogenic and pro-inflammatory cytokines (IL-8, GDF-15, MCP-1, MMP-9, PTX-3, and THBS-1). Due to the low number of cytokines evaluated by this method, further analysis (such as miRNA content evaluation and proteomic studies) should be performed to better understand the influence of a different culture system on secretome composition.

EVs derived from different culture conditions were also isolated and characterized. Surprisingly, culture conditions also affected the release of EVs, a cell product extremely important in the creation of cell-free therapeutic approaches. In this study, we demonstrated that MSC spheroid culture stimulates a higher release of sEVs compared to the 2D culture, showing the double advantage of delaying cellular senescence onset and easily obtaining a higher amount of EVs. sEVs maintain classical phenotypical characteristics, particularly in terms of tetraspanin expression, which are necessary molecules for sEV biogenesis (Raposo and Stoorvogel, 2013).

Studies on MSC-derived EVs are particularly focused on the creation of therapeutic tools for the treatment of inflammatory-related disorders, such as osteoarthritis and autoimmune diseases (Palamà et al., 2020). However, the use of MSC-derived EVs in the treatment of vasculogenesis-related disorders is not so exploited. In this context, the biological effect of different secretome and EV fractions derived from different culture conditions was investigated using an *in vitro* HUVEC angiogenic assay. According to secretome analysis, large spheroids-derived soluble factors (CM and DM) showed a stronger effect on HUVEC migration and tube formation compared to the 2D control. However, the potential was limited compared to the soluble factors (CM and DM) derived from small spheroids, which also exhibited a strong effect on HUVEC proliferation induction. The weaker pro-angiogenic effect of large spheroid-derived soluble factors could be explained by the presence of strong anti-angiogenic factors, highlighted by secretome analyses. These inhibitory factors were indeed downregulated in small-sized spheroids, allowing them to exert a higher angiogenic potential.

We also observed that small spheroid-derived sEVs possess an enhanced angiogenic potential capable of stimulating all the key parts of angiogenesis, while no significant effects were observed for large spheroid-derived sEVs. Regarding the different effects of secretome fractions on HUVECs, we suggest a synergic action of soluble factors and EVs derived from 3D culture in the stimulation of the different phases of the angiogenic process (Gorgun et al., 2021). This hypothesized synergism was confirmed by the evidence that the fraction enriched in only soluble factors (DM) and EV population alone have a minor effect compared to the complete conditioned medium.

Considering all the data together, spheroids represent a possible alternative culture system that may be used for the maintenance of MSCs. This study demonstrated the relevance of the size of the generated spheroids to avoid the generation of a hypoxic core that will lead to an increase in cellular senescence and an alteration in the MSC secretory profile. In particular, MSCs present in small spheroids maintain the classical phenotype and show reduced cellular senescence. Moreover, MSC spheroid secretome is enriched in pro-angiogenic cytokines, suggesting a potential use of small spheroid-derived soluble factors and sEVs as a cell-free therapeutic tool for the treatment of vasculogenesis-related disorders (myocardial infarction or stroke), in which the creation of new vessels after the traumatic event is needed (Rani et al., 2015; Todorova et al., 2017; Ferreira et al., 2018; Praveen Kumar et al., 2019; Rezaie et al., 2019).

Overall, MSC spheroids are known to display increased paracrine secretion, proliferation, stemness, and anti-inflammatory and pro-angiogenic properties compared to the 2D condition. Furthermore, several studies indicate that MSC spheroid bioprinted in a biomaterial significantly supports MSC functionality and improves MSC engraftment in damaged tissues, increasing MSC survival *in vivo*. On this basis, the development of a reproducible large-scale manufacturing protocol to produce MSC spheroids would promote their use in clinical and therapeutic applications (Murphy et al., 2017; Allen et al., 2019; Almeida et al., 2020; Yuan et al., 2022).

## Data Availability

All relevant data supporting the findings of this study are available within the paper, its supplementary information and from the corresponding authors upon reasonable request.
