# Supplementary material for: Spheroid size influences cellular senescence and angiogenic potential of mesenchymal stromal cell-derived soluble factors and extracellular vesicles
Source: Front Bioeng Biotechnol. 2023 Dec 12;11:1297644. doi: 10.3389/fbioe.2023.1297644 (PMC10756914; doi:10.3389/fbioe.2023.1297644)
Supplement: Supplementary file 1 [file DataSheet1.pdf]

Supplementary Material

Spheroid size influence cellular senescence and angiogenic potential of mesenchymal stromal cells derived soluble factors and extracellular vesicles

Rovere M[1], Reverberi D[2], Pietro Arnaldi[1], Palamà M.E.F[1], Gentili C[1*]

*** Correspondence:** Chiara Gentili: chiara.gentili@unige.it

1 Supplementary materials

1.1 Osteogenic differentiation

To test osteogenic differentiation, 2D cells were seeded in 24-well plates at a density of 10^5 cells/cm^2 while spheroids were formed as explained in paragraph 2.2 of the manuscript. MSC were cultured in the presence of osteogenic induction medium containing 5 µg/mL ascorbic acid, 10^{-7} mol/L dexamethasone and 10 mmol/L β glycerophosphate (all from Sigma-Aldrich, St. Louis, MO, USA). A 2D control was maintained in complete medium as negative control. After 3 weeks of culture, RNA from the different conditions were extracted and a quantitative real-time polymerase chain reaction was performed to evaluate the expression of *RUNX2*, *ALP*, *OCN*, *COL1A*, *Osterix* (for more detail of the RNA extraction and qRT-PCR protocol see paragraph 2.7 on the manuscript).

2 Supplementary Figures

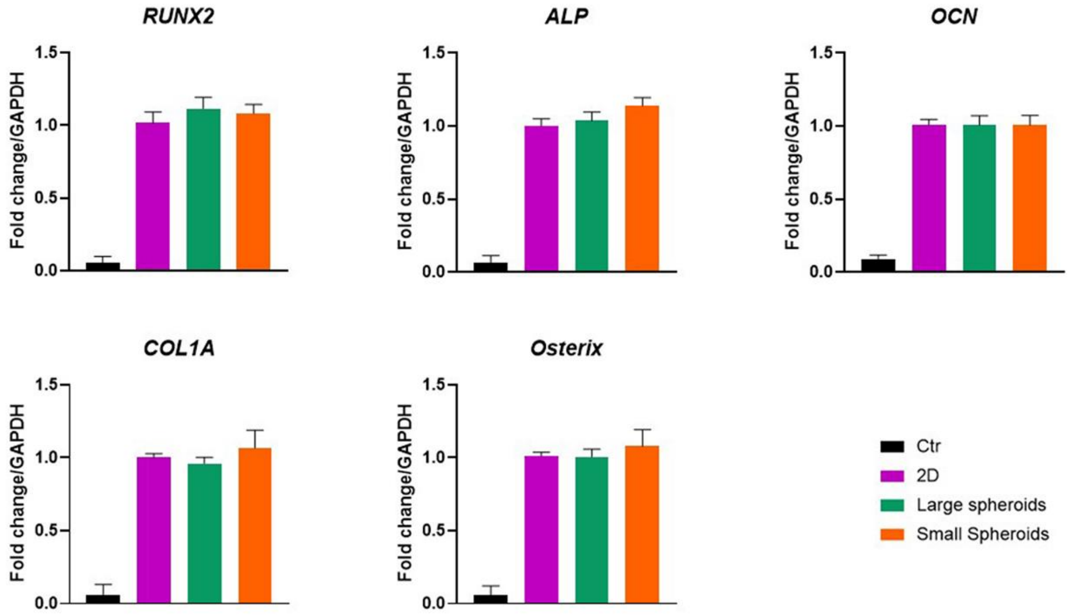

Supplementary Figure 1. Osteogenic differentiation of MSC spheroids. Quantitative Real Time PCR for the expression of *RUNX2:* RUNX family transcription factor 2, *ALP:* alkaline phosphatase, *OCN:* osteocalcin, *COL1A:* collagen type 1 alpha, *Osterix*, data are represented as mean ± SD. (N=3, One-way Anova).

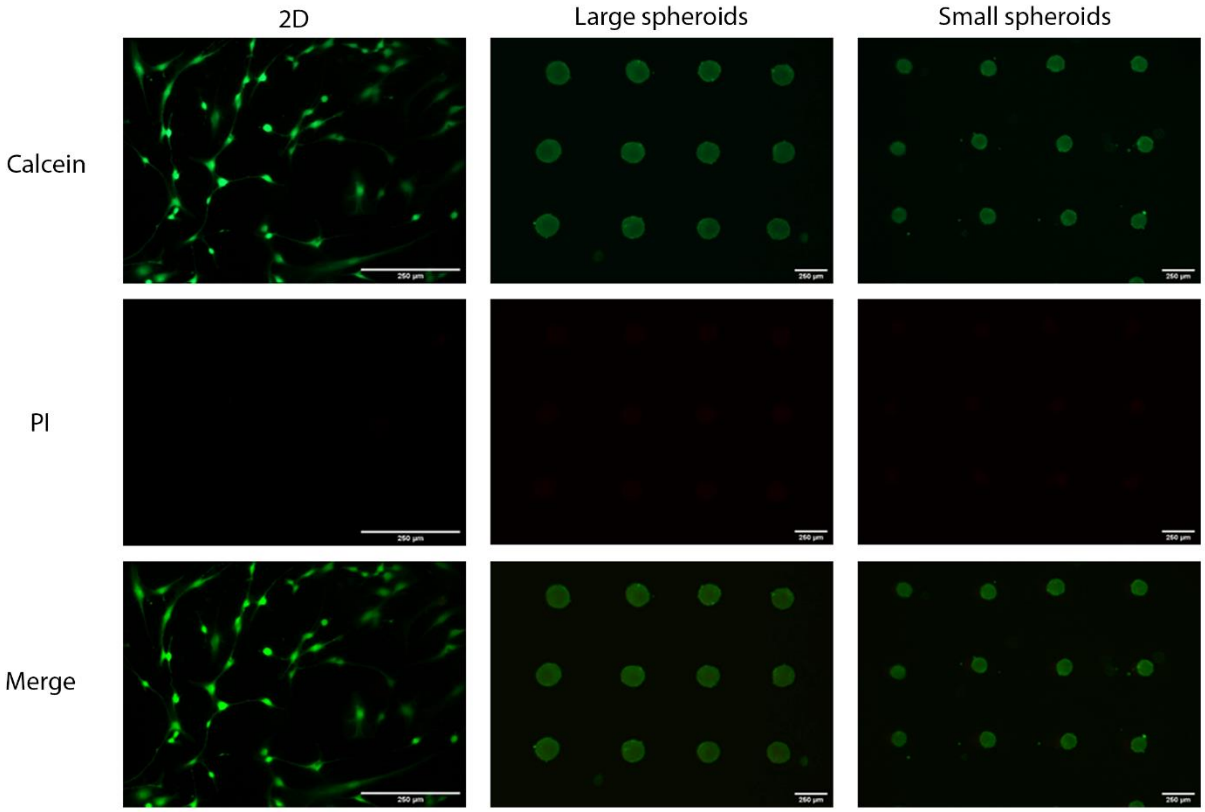

Supplementary Figure 2. LIVE/DEAD viability assay. Representative images of LIVE/DEAD viability assay on different culture conditions. From the top pictures show live cells (calcein), dead cells (PI) and merge (N=3), scalebar = 250 µm.

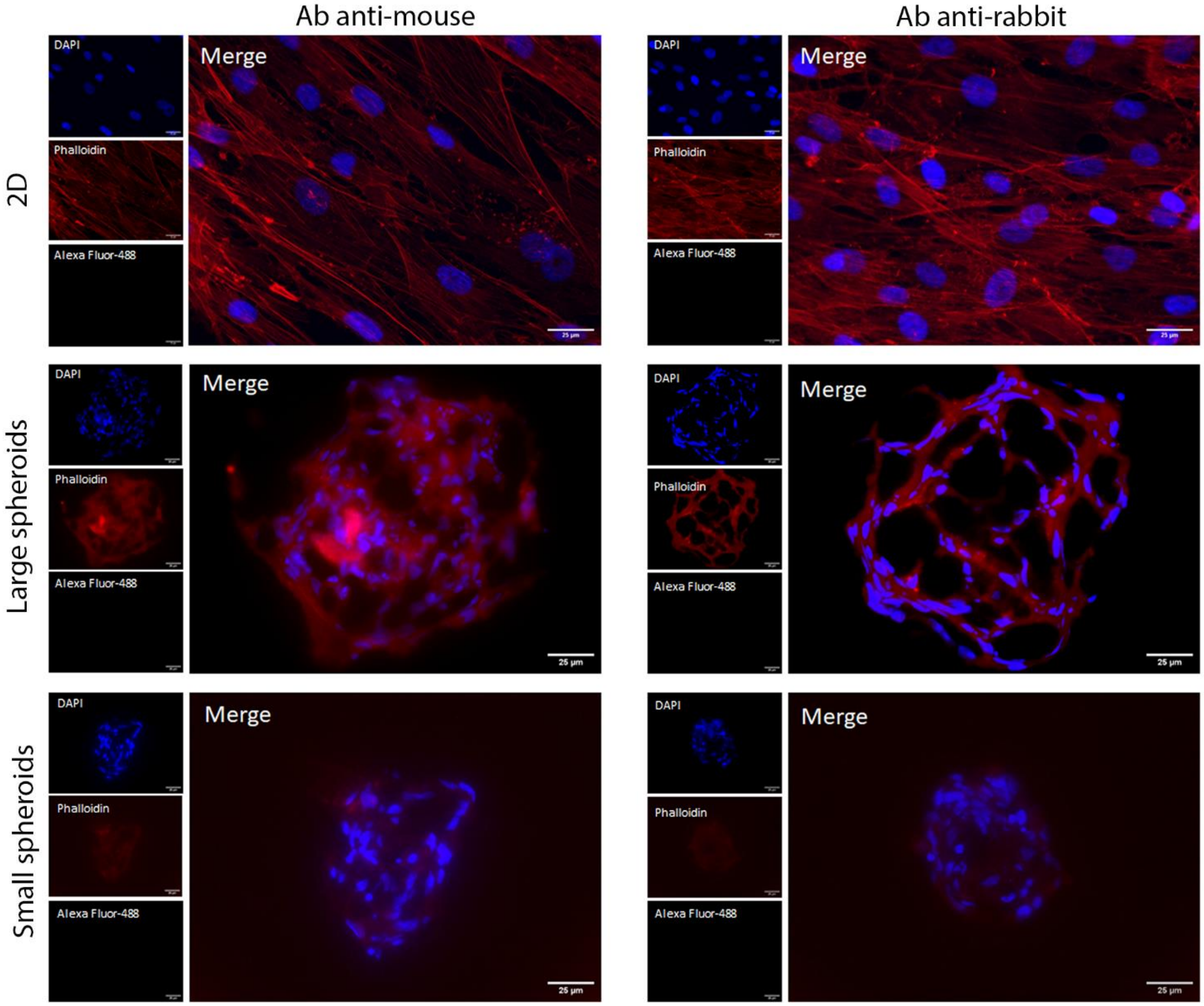

Supplementary Figure 3. Non-specific binding of secondary antibody in immunofluorescence staining assessment. Representative images of control for aspecific binding of secondary antibody used in immunostaining. From the top images show nuclei (DAPI), Phalloidin, secondary antibody aspecific binding (Alexa fluor-488) and merge, scalebar = 25 µm.

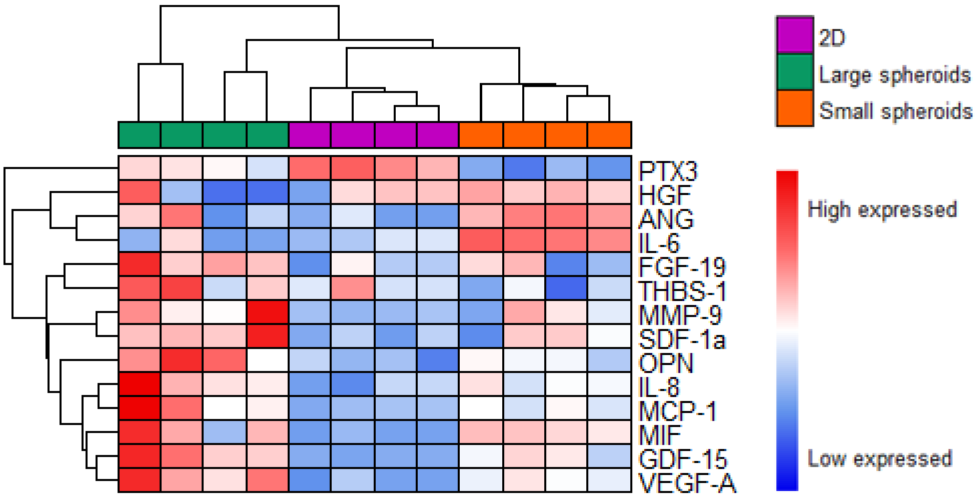

Supplementary Figure 4. Proteome profiling analisys of MSCs secretome. Heatmap of modulated proteins on cytokine array of CM derived from 2D, Big and Large spheroids culture of MSCs. Color code identify normalized pixel intensity (N=4). ANG: Angiogenin, FGF-19: Fibroblast Growth Factor 19, GDF-15: Growth Differentiation Factor 15, IL-6: Interleukin 6, IL-8: Interleukin 8, MCP-1: Monocyte Chemoattractant protein 1, MIF: Macrophage Migration Inhibitory Factor, MMP-9: Matrix Metallopeptidase 9, OPN: Osteopontin, PTX3: Pentraxin 3, SDF1a: Stromal Cell-derived factor 1, THBS-1: Thrombospondin 1, VEGF-A: Vascular Endothelial Growth factor-A, HGF: Hepatocyte Growth Factor.

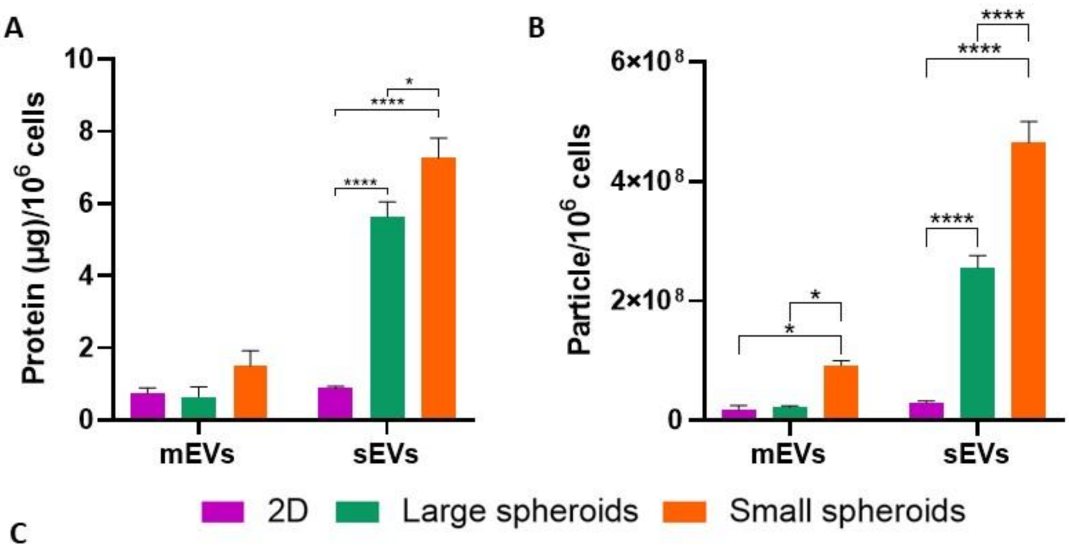

C

	Mean size	Peak size
2D mEVs	159.34 ± 12.67 nm	153.23 ± 19.34 nm
2D sEVs	129.56 ± 9.43 nm	118.67 ± 11.92 nm
Large spheroids mEVs	162.45 ± 22.44 nm	148.78 ± 23.68 nm
Large spheroids sEVs	131.56 ± 9.34 nm	116.98 ± 8.34 nm
Small spheroids mEVs	156.68 ± 16.23 nm	146.78 ± 14.67 nm
Small spheroids sEVs	122.68 ± 4.43 nm	113.78 ± 15.27 nm

Supplementary figure 5. BCA and NTA characterization of EVs isolated after 120 hours of culture. (A) Quantification of protein content on mEVs and sEVs by BCA assay, data are normalized on cells number (µg/10^6 cells). Data are represented as mean ± SD, * p-value < 0.05, **** p-value < 0.0001 (N=3, Two-way Anova and Tukey multiple comparison). **(B)** NTA of particle concentration, expressed as particle/10^6 cells. Data are represented as mean ± SD, * p-value < 0.05, **** p-value < 0.0001 (N=3, Two-way Anova and Tukey multiple comparison). **(C)** Table show NTA analysis of size of mEVs and sEVs isolated from 2D, Large and Small spheroids. Data are reported as mean ± SD (N=3).

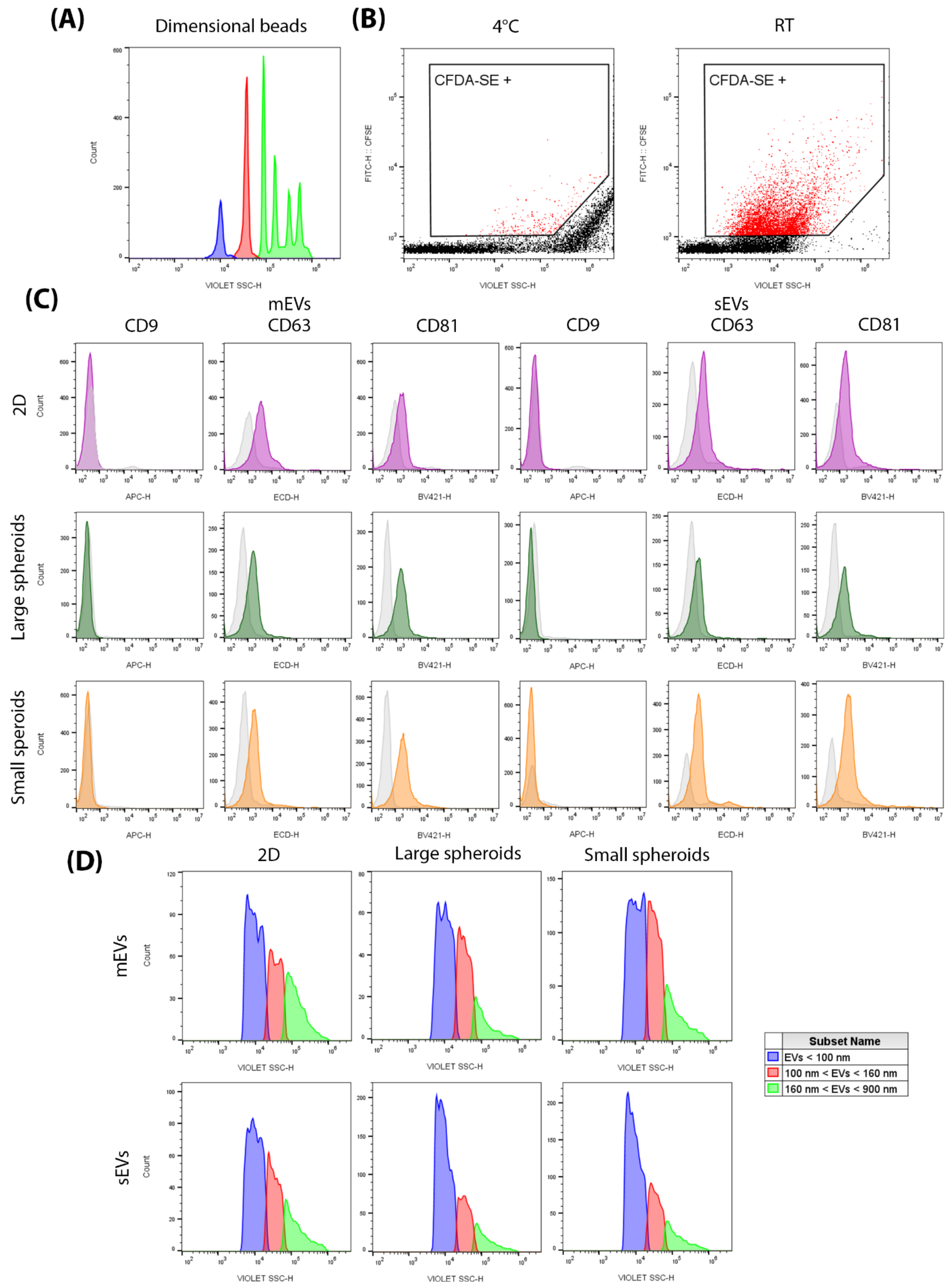
(A)
Dimensional beads
Count
VIOLET SSC-H
(B)
4°C
RT
CFDA-SE +
FITC-H :: CFSE
VIOLET SSC-H
(C)
mEVs
sEVs
CD9
CD63
CD81
2D
Large spheroids
Small speroids
Count
APC-H
ECD-H
BV421-H
(D)
2D
Large spheroids
Small spheroids
mEVs
sEVs
Count
VIOLET SSC-H
Subset Name
EVs < 100 nm
100 nm < EVs < 160 nm
160 nm < EVs < 900 nm

Supplementary Figure 6. Non-conventional flow-cytometry analysis for EVs characterization. (A) Representative histogram of fluorescent beads. **(B)** Representative CFDA-SE staining used to identify intact vescicles. EVs were stained with CFDA-SE at 4°C as control (left) to define the appropriate dimensional gate considering the EVs stained with CDFA-SE at room temperature (right). Red areas identify CFDA-SE positive events. **(C)** Representative histograms reporting flow cytometry analysis of MSCs cultured in 2D (purple), Large (green) or Small (orange) spheroids. Histogram curves identify cells reacting with CD9, CD63, CD81 specific antibodies. Area under the gray curve identify the reaction of the cells with the correspondent non-reactive immunoglobulin of the same isotype (isotype control). Data are representative of three independent experiments. **(D)** Size distribution analysis of EVs, for the analysis were defined three dimensional gates with dimensional beads: EVs < 100 nm (blue), 100 nm < EVs < 160 nm (red) and 160 nm < EVs < 900 nm (green), used to identify the percentage of the different subtypes of EVs present in each gate. Presented data are from one representative experiment.

3 Supplementary tables

Supplementary table 1 Primer used for quantitative real-time PCR for osteogenic differentiation.

Gene	Forward primer	Reverse primer
GAPDH	5’-CCATCTTCCAGGAGCGAGAT-3’	5’- CTGCTTCACCACCTTCTTGAT-3’
RUNX2	5’-ATGAGAGTAGGTGTCCCGCC-3’	5’- GGGGTGGTAGAGTGGATGGA -3’
ALP	5’- GTATGAGAGTGACGAGAAAGCC-3’	5’-GTTCCAGATGAAGTGGGAGTG-3’
OCN	5’-TTCTTTCCTCTTCCCCTTG-3’	5’-CCTCTTCTGGAGTTTATTTGG-3’
COL1A	5’-AGGGCTCCAACGAGATCGAGATCCG-3’	5’-TACAGGAAGCAGACAGGGCCAACG-3’
Osterix	5’-TGGGCTCCCAACACTATTTC-3’	5’-GGGAAGACTGAAGCCTGGA-3’

Supplementary table 2. Spheroids size variation during culture time. Table report size variation of Large and Small spheroids during culture time. Diameter was calculated on 100 spheroids for each time-point and for each replicate, by using ImageJ. Data are represented as mean ± SD (N=3).

	24 h	48 h	72 h	96 h	120 h	144 h	168 h	192 h
**Large spheroids**	427.20 ± 30.42 µm	391.87 ± 21.62 µm	365.37 ± 27.67 µm	339.52 ± 19.71 µm	288.64 ± 34.63 µm	295.63 ± 21.00 µm	284.96 ± 30.89 µm	285.22 ± 24.20 µm
**Small spheroids**	268.17 ± 15.16 µm	218.69 ± 12.76 µm	198.95 ± 16.86 µm	194.29 ± 20.78 µm	209.33 ± 20.05 µm	203.5 ± 18.52 µm	209.33 ± 18.76 µm	206.23 ± 17.15 µm

Supplementary table 3. Spheroids cell number analysis. Table report the number of cell composing the spheroids after 72 hours of culture calculated after digestion of the aggregate. Data are represented as mean ± SD (N=3).

	Large spheroids	Small spheroids
**Spheroid cell number**	2592 ± 125	1032 ± 174
